# Biomonitoring of Inorganic Pollutants in Blood Samples of Population Affected by the Tajogaite Eruption: The ISVOLCAN Study in Spain

**DOI:** 10.3390/toxics13070581

**Published:** 2025-07-10

**Authors:** Katherine Simbaña-Rivera, María Cristo Rodríguez-Pérez, Manuel Enrique Fuentes-Ferrer, Manuel Zumbado Peña, Ángel Rodríguez Hernández, Julia Eychenne, Lucie Sauzéat, Damary S. Jaramillo-Aguilar, Ana Rodríguez Chamorro, Luis D. Boada

**Affiliations:** 1 Toxicology Unit, Research Institute of Biomedical and Health Sciences (IUIBS), University of Las Palmas de Gran Canaria (ULPGC), 35016 Las Palmas de Gran Canaria, Spain; manuel.zumbado@ulpgc.es (M.Z.P.); luis.boada@ulpgc.es (L.D.B.); 2 ECUAVOLCAN Research Group, Faculty of Medicine, Pontificia Universidad Católica del Ecuador (PUCE), Quito 170143, Ecuador; damarysjaramillo@gmail.com; 3 Research Unit, University Hospital Nuestra Señora de Candelaria and Primary Care Authority of Tenerife, Canary Health Service, 38010 Santa Cruz de Tenerife, Spain; cristi_rodriguez@yahoo.es (M.C.R.-P.); mfuentesferrer@gmail.com (M.E.F.-F.); 4 Preventive Medicine Department, University Hospital Nuestra Señora de Candelaria, Canary Health Service, 38010 Santa Cruz de Tenerife, Spain; 5 Toxicology Unit, Department of Biomedical Sciences, Universidad de Alcalá, 28801 Alcalá De Henares, Spain; angel.rodriguezh@uah.es; 6 Laboratoire Magmas et Volcans, IRD, CNRS, OPGC, Université Clermont Auvergne, F-63000 Clermont-Ferrand, France; julia.eychenne@uca.fr (J.E.); lucie.sauzeat@uca.fr (L.S.); 7 Institut de Génétique Reproduction et Développement, Université Clermont Auvergne, CNRS, INSERM, F-63000 Clermont-Ferrand, France; 8 Primary Care Authority of Tenerife, Canary Health Service, 38010 Santa Cruz de Tenerife, Spain; archadc@gmail.com

**Keywords:** biomonitoring, volcanic eruptions, inorganic pollutants, environmental exposure, environmental toxicology

## Abstract

Volcanic eruptions release gases and particulates that may adversely affect human health. The Tajogaite eruption on La Palma provided a unique opportunity to evaluate inorganic pollutant exposure in a directly affected population. As part of the ISVOLCAN study, blood samples from 393 adults residing in the island’s western region were analyzed for 43 inorganic elements using Inductively Coupled Plasma Mass Spectrometry (ICP-MS), including 20 toxic elements identified by the Agency for Toxic Substances and Disease Registry (ATSDR). The median age of participants was 51 years, and 56.7% were female. Higher levels of Hg and Mn were associated with long-term occupational exposure, while smoking was linked to elevated Cd, Pb, and Sr levels. Participants living within 6.5 km of the volcano had significantly higher concentrations of Al and Ti. Ash cleanup activities were associated with increased levels of Ni and Cu, and those spending over five hours outdoors daily showed elevated Se and Pb. This is the first biomonitoring study to assess blood concentrations of inorganic pollutants in a population exposed to volcanic emissions. The findings highlight key exposure factors and underscore the need for continued research to assess long-term health effects and inform public health measures.

## 1. Introduction

Volcanic eruptions are natural hazards that can have significant consequences on human health due to the release of gases and particles into the ambient air [1,2]. Volcanic ash, the particles formed by the magma fragmentation in explosive eruptions, is a major source of environmental contamination. Ash is rich in heavy metals, and, depending on their grain size, it can be inhaled or ingested by nearby populations. The physicochemical properties of these material play a crucial role in determining the associated health risks [3,4]. They have been linked to a variety of acute health issues, particularly affecting the respiratory system and causing ocular alterations [5,6]. In vitro and in vivo studies also demonstrated that ash exposure triggers a weak pro-inflammatory response in alveolar epithelial cells [7,8], and induces significant metallome deregulations at the whole-body scale associated with pathophysiological alterations, as demonstrated in a mouse model [9]. Volcanic gas contains major species such as H_2_O, SO_2_, CO_2_, HCl, and HF, as well as metal pollutants in significant amounts [10,11], also known to cause acute health issues [12,13]. Additionally, the limited studies that have evaluated the effects of prolonged exposure to volcanic gas emissions have demonstrated an association with an increased risk of respiratory system disorders [14].

Volcanic emissions contain inorganic contaminants that can accumulate in the environment and human body. These include lead (Pb), mercury (Hg), cadmium (Cd), arsenic (As), chromium (Cr), cobalt (Co), manganese (Mn), nickel (Ni), copper (Cu), zinc (Zn), and iron (Fe), some of which are known for their toxic effects on human health [15]. Studies in volcanic regions have shown that populations exposed to volcanic activity have significantly higher levels of certain inorganic elements compared to those in non-volcanic areas [16]. Furthermore, residents of active volcanic areas exhibit elevated concentrations of inorganic toxicants in their urine and hair, indicating complex non-anthropogenic biocontamination [16].

The Agency for Toxic Substances and Disease Registry (ATSDR) has identified up to 20 elements that require monitoring due to their effects on human and environmental health, based on their frequency, toxicity, and potential for human exposure [17]. Notably, this list includes essential elements for normal bodily functions; however, exposure to volcanic emissions could alter their levels in the body and pose potential health risks [18].

The eruption of the Tajogaite volcano on La Palma, Spain, began on 19 September 2021, and lasted for 85 days. This event released large quantities of volcanic emissions containing various inorganic contaminants over several weeks, significantly impacting air quality, water sources, and soil composition [19,20].

La Palma is the fifth most densely populated island of the Canary Islands, with nearly half of its population residing in municipalities near the volcanic eruption site [21]. The island’s diverse topography and climatic conditions foster a population dependent mainly on agriculture and tourism. The deposition of volcanic ash and contamination of ambient air by volcanic ash and gases in these areas not only disrupted economic activities, but also raised concerns about the long-term health impacts on residents. This study aims to describe the inorganic toxicant levels in blood samples collected from adult participants in the ISVOLCAN study (Health Impact on the Population of La Palma caused by the Tajogaite Volcano Eruption) residing in the western region of La Palma and to analyze the variations in these levels according to sociodemographic characteristics and level of exposure during the volcanic eruption.

## 2. Materials and Methods

### 2.1. Study Design and Participants

The ISVOLCAN study [22] is a prospective observational cohort study conducted among a randomly selected general population across municipalities in the western region and the eastern region of La Palma Island. The study is structured in two phases: the initial phase, spanning from 2022 to 2023, involved the recruitment of participants, administration of an epidemiological questionnaire via telephone, and a health center visit for physical examinations, pulmonary function tests, and venous blood extraction. These samples were used for subsequent studies involving clinical and toxicological laboratory parameters. The subsequent phase will monitor the cohort at intervals of 2, 5, and 10 years. This study presents an analysis of blood test results from the initial 393 samples collected from general population residing in the island’s western region during the volcanic eruption, which is the most exposed population to metal-rich volcanic emissions. These participants constitute 59.4% of the total study cohort from that specific geographic area. Sex and gender were considered in the study design by ensuring the inclusion of both male and female participants without restriction.

### 2.2. Study Area

Spain’s geographic diversity is expressed through its continental and insular regions. The continental territory, predominantly located on the Iberian Peninsula, constitutes the bulk of Spain’s area. The insular territory includes the Balearic Islands in the Mediterranean Sea and the Canary Islands off the northwest coast of Africa. The Canary Islands’ unique microclimates, endemic species, Saharan dust, and traditional practices—like gofio and seafood consumption—affect metal bioavailability and baseline exposure to elements such as aluminium, cadmium, lead, strontium, and mercury [23,24], with additional influences from soil contact and local crop intake in volcanic rural settings [25].

Among the Canary Islands, La Palma is distinguished by its rich biodiversity and varied landscape. Located at 28°26′ N latitude and 14°01′ W longitude, the island covers approximately 708 km^2^ and has a population of over 83,000 inhabitants. In the western region near the volcano, about 41,000 residents are concentrated [26].

**Figure 1:**
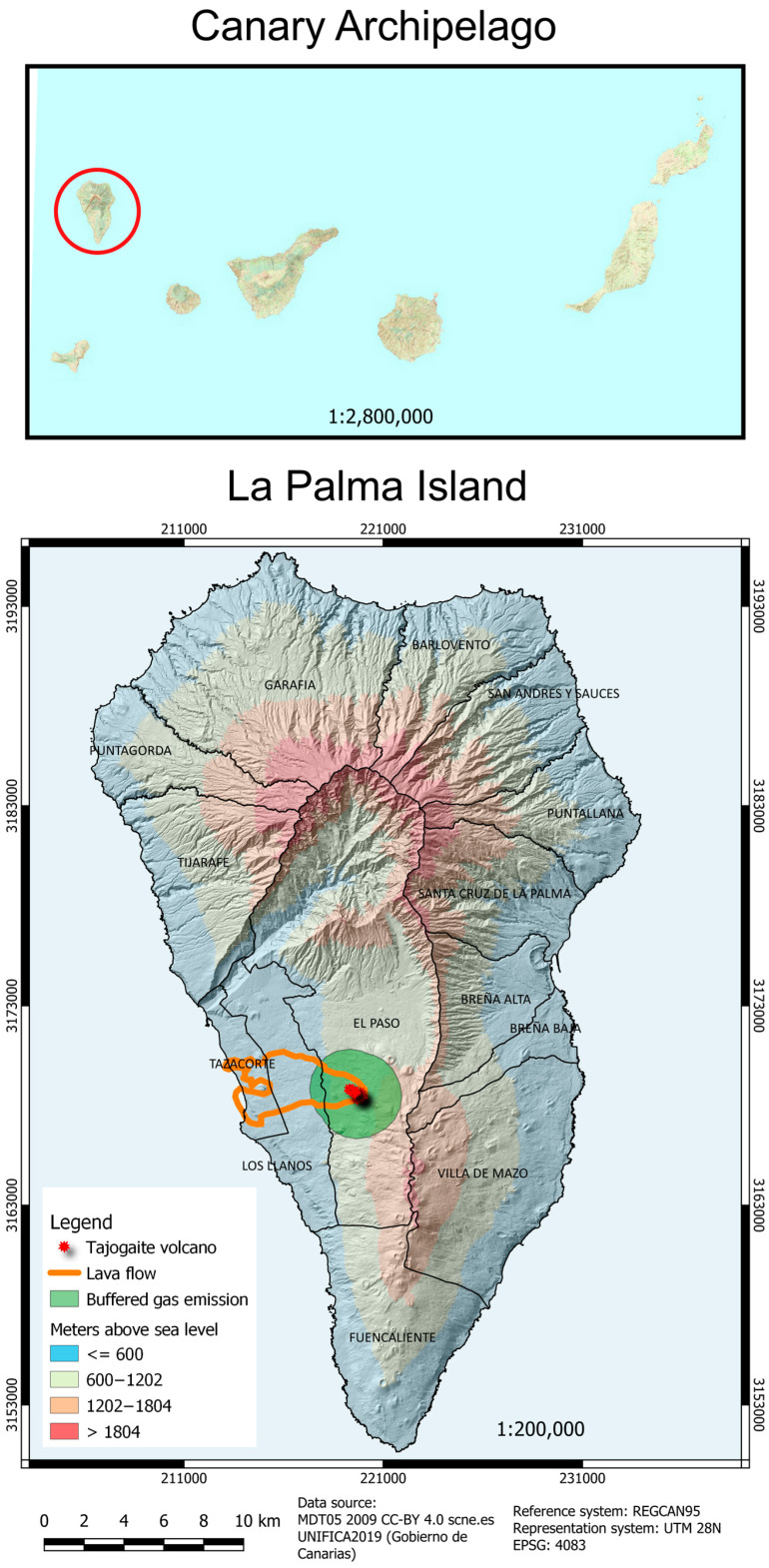
Location of La Palma Island in the Canary archipelago (red circle) and geography of La Palma, showing the location of the different districts, the location of the Tajogaite Volcano, the buffered gas emission, and the extent of the lava flows (figure generated with QGIS Development Team 2.8).

In 2021, La Palma witnessed a significant volcanic event in the Cabeza de Vaca area on the western slope of the Cumbre Vieja ridge, within the El Paso municipality. This long-lasting and hybrid eruption culminated in the formation of the Tajogaite volcano, which reached a peak elevation of 1131 m above sea level. The eruption showcased a variety of volcanic activities, including lava flow emplacements, lava fountaining, and Strombolian explosions that produced sustained ash and gas emissions [27]. These activities predominantly impacting the Valle de Aridane with considerable damage from lava, gases, and particulate matter [28] (Figure 1).

### 2.3. Data and Blood Sample Collection

For this study, the variables analyzed from the epidemiological questionnaire (available at www.estudioisvolcan.com) included sociodemographic factors such as age, sex, educational level, employment status, and occupation type (classified according to the European Commission’s framework) [29]. Additionally, variables regarding occupational exposure to toxic substances, types of toxic substances (classified according to the ATSDR’s Toxic Substance Classification) [30], duration of exposure to toxic substances, smoking exposure, smoking status, and duration of smoking exposure were also recorded.

Variables concerning the level of volcanic exposure assessed included distance to the volcano in kilometers, volcanic ash cleaning activities, location of cleaning, tools used, cleaning frequency, daily hours spent outdoors, frequency of mask usage outdoors, and frequency of protective eyewear usage outdoors.

Blood samples were collected from participants during their visit to the health center in Los Llanos de Aridane. These samples were daily transported and stored at the Laboratory of the University Hospital of La Palma (Canary Islands, Spain). They were then shipped weekly to the Research Unit of the Hospital Nuestra Señora de Candelaria in Tenerife and stored at −80 °C for whole blood samples. The toxicological analyses including the measurement of inorganic elements were conducted at the Toxicology Unit in the Research Institute of Biomedical and Health Sciences (IUIBS) at the University of Las Palmas de Gran Canaria (Canary Islands, Spain).

### 2.4. Inorganic Element Measurements 

The analysis incorporated 43 elements, including 20 identified from the ATSDR’s Substance Priority List of 2023 [17] chosen for their prevalent toxicity and potential exposure risk to populations. This comprehensive selection also included elements previously not characterized as harmful or prioritized as contaminants. Specifically, rare earth elements (REEs) and other minority elements (MEs), now recognized as emerging pollutants, are released into the environment during volcanic eruptions and are accumulating over time in global populations. The full spectrum of inorganic elements analyzed comprised highly toxic elements: Arsenic (As), Cadmium (Cd), Mercury (Hg), and Lead (Pb); potentially toxic elements: Aluminium (Al), Barium (Ba), Beryllium (Be), Chromium (Cr), Caesium (Cs), Nickel (Ni), Antimony (Sb), Strontium (Sr), Thorium (Th), Thallium (Tl), Uranium (U), and Vanadium (V); essential elements with potential toxicity under undesired exposure: Cobalt (Co), Copper (Cu), Manganese (Mn), and Selenium (Se); and REEs and other MEs seldom studied in the context of volcanism: Bismuth (Bi), Cerium (Ce), Dysprosium (Dy), Erbium (Er), Europium (Eu), Gadolinium (Gd), Holmium (Ho), Indium (In), Lanthanum (La), Lutetium (Lu), Niobium (Nb), Neodymium (Nd), Praseodymium (Pr), Platinum (Pt), Rubidium (Rb), Ruthenium (Ru), Samarium (Sm), Tin (Sn), Terbium (Te), Titanium (Ti), Thulium (Tm), Yttrium (Y), and Ytterbium (Yb).

The methodology for this study was adapted from previously established protocols by our research group as detailed by Henríquez-Hernández in 2023 [31]. Specifically, 0.5 mL of whole blood samples was processed by the protocol for acid digestion. The internal standard (ISTD) solution, containing scandium (Sc), germanium (Ge), rhodium (Rh), and iridium (Ir) at concentrations of 20 mg/mL each, was introduced into the ICP-MS at a concentration of 40 ppb. This step was undertaken to evaluate the reproducibility of counts per seconds of the ions (CPS) and associated relative standard deviation (RSD) values, as well as the recovery rates of the four isotopes (^45^Sc, ^72^Ge, ^103^Rh, ^193^Ir), which should range between 70% and 130%. This was introduced using an Agilent 7900 ICP-MS system (Agilent Technologies, Tokyo, Japan), equipped with standard nickel cones, a MicroMist glass concentric nebulizer, and an Ultra High Matrix Introduction (UHMI) system. The 4th generation Octopole Reaction System (ORS4) operated in helium (He) mode for all elements to minimize interferences from low-mass elements, enhancing detection limits for most elements. Elemental standards were obtained as certified reference material from CPA Chem (Stara Zagora, Bulgaria), either as multi-elemental or individual acid solutions. Calibration procedures were meticulously designed for optimal quantification, involving three different calibration curves: (1) a multi-elemental curve covering major heavy metals and some minority elements (nine points from 0 to 2000 ppb); (2) a mercury curve (five points from 0 to 50 ppb); and (3) a curve for REEs and the majority of MEs (six points from 0 to 25 ppb), based on their linearity, sensitivity, and precision. To prevent memory effects, especially with mercury, a cleaning solution containing 2.0% HNO3 and 1% HCl was used between each analysis. Each sample was analyzed in three separate vials, with three complete readings automatically performed from each, providing nine individual measurements per sample. Element quantification was conducted using the MassHunter v.4.2 ICP-MS Data Analysis software by Agilent Technologies. Before samples determination, the entire procedure underwent in-house validation using a certified reference material (CRM, Seronorm™ Trace Elements Whole Blood, Billingstad, Norway) at three concentration levels (L-1, L-2, L-3). The RCM comprised three clinically relevant levels for all elements studied, except In and Ti, which were assessed with fortified matrix assays. 

Calibration curves generally displayed excellent regression coefficients (≥0.995), and recoveries ranged from 79% to 121% for REEs and MEs, and from 76% to 124% for ATSDR’s toxic elements. Limits of detection (LODs) and quantification (LOQs) were determined based on signals significantly above the noise level of the blank solution (pure acids). The LOD and LOQ values for each analyte, along with precision and accuracy data from CRM and spiked samples, are provided in Supplementary File S1. As a quality control for blood sample analysis, independent vials were included every 24 vials of sample replicates in the sequence, each containing: a digested blank, the two customized mixes, and each level (L-1, L-2, and L-3) of the CRM. This was performed to verify potential contaminations and ensure an accurate and replicable determination (results were acceptable when the analyte concentration fell within 20% of the RSD value).

### 2.5. Statistical Analysis

Qualitative variables will be described by displaying both their absolute and relative frequencies. Continuous variables were summarized using median values and interquartile ranges (IQRs) or mean and standard deviation, as appropriate, depending on their distribution as determined by the Kolmogorov–Smirnov test. Differences between sociodemographic factors or variables related to the level of volcanic exposure and the concentration of inorganic pollutants present in more than 30% of blood samples were analyzed using the Mann–Whitney and Kruskal–Wallis tests.

Bivariate correlations for continuous variables were assessed using Spearman’s correlation tests. Statistical significance was defined as a probability level of <0.05 (two-tailed).

All statistical analyses were conducted using R Core Team software 2018 version 3.5.1. 

## 3. Results

### 3.1. Sociodemographic Characteristics

The study included 393 participants from the western region of La Palma with a median age of 51 years (IQR: 40–59), and 56.7% females. More than half of the participants lived within 6.5 km of the volcano. Educational levels varied, with 47.1% having secondary education and 24.4% holding a university degree. Employment status during the eruption indicated most people were employed, and occupational exposure to toxic substances was reported by 17.8% of participants, which primarily involved solvents and vapors. Among the participants, most had comorbidities, and over 44% were exposed to smoking (current and former smokers). Regarding volcanic ash cleaning, around 92% engaged in these activities, being mostly ash cleaning both in indoor and outdoor environments. Most people used high particle projection tools, such as brooms and blowers, and 47.3% cleaned at least once daily. Outdoor exposure was significant, with 51.4% spending more than five hours daily outside. Protective measures included frequent mask use (over 84%) and protective eyeglasses (57.5%) (Table 1, Supplementary Table S1).

### 3.2. Measurement of Inorganic Elements

In the analysis of environmental toxicants (Table 2, Supplementary Table S2), Cu and Se were identified as the most prevalent inorganic elements, detected in 100% of the samples. These elements exhibited median concentrations of 842.3 ng/mL for Cu and 109.2 ng/mL for Se. Rb was also prominent, present in all samples and displaying the highest median concentration of 1848.8 ng/mL. Similarly, Mn and Hg showed widespread presence, detected in 96.4% and 98.7% of the samples, respectively. Pb was found in a significant proportion of samples, with a mean concentration of 7.7 ng/mL and a median of 6.0 ng/mL. Al, though detected in only 30.5% of samples, had a notably high median concentration of 80.4 ng/mL (IQR: 39.4–180.8 ng/mL). Cd and Co, with detection frequencies above 44%, indicated considerable exposure among the study participants. In contrast, Tl and Tb were not detected, suggesting minimal exposure to these elements. Additionally, U and V were detected at relatively low rates of 1.5% and 2.8%, respectively.

### 3.3. Inorganic Contaminants and Their Association with Sociodemographic Characteristics

Table 3 (Supplementary Table S3) displays the distribution of 14 inorganic pollutants, each detected in over 30% of the sample population. This distribution is analyzed in relation to various sociodemographic variables. Notably, the median concentrations of heavy metals such as Pb, Hg, and Cd exhibit significant variations based on sex, age, and educational level, respectively. Individuals over the age of 50 demonstrated higher median levels of Hg (3.6 ng/mL) and Sr (14.9 ng/mL) compared to their younger counterparts. Additionally, males demonstrated significantly elevated levels of Pb and Rb, with median concentrations of 8.7 ng/mL and 1956.3 ng/mL, respectively. Conversely, females had higher concentrations of Cu (913.97 ng/mL), Al (100.07 ng/mL), and Ni (4.40 ng/mL) compared to males.

In terms of educational attainment, individuals with only elementary education showed increased levels of Hg, with a median of 3.3 ng/mL (*p* < 0.01). Employment status during the eruption also influenced exposure levels, with employed individuals presenting lower median levels of Cd than those who were unemployed. Moreover, individuals with occupational exposure to toxic substances did not show significant differences in the concentrations of various inorganic elements.

Duration of occupational exposure to toxic substances demonstrated significant association with the median concentrations of inorganic elements. Specifically, levels of Hg, Mn, and Cu varied notably among individuals with more than 15 years of occupational exposure to toxic substances. Current smokers exhibited significantly higher levels of Cd compared to non-smokers and ex-smokers, and those with smoking histories exceeding 20 years displayed elevated levels of metals such as Pb and Sr.

### 3.4. Inorganic Pollutants and Their Association with Level of Exposure to Volcano

When comparing volcanic exposure based on residential distance, it was observed that individuals living within 6.5 km of the volcano had slightly higher median concentrations of Al (*p* = 0.034) and Ti (*p* = 0.032). In contrast, those residing beyond 6.5 km exhibited elevated levels of Se and Sn, with median concentrations of 111.5 ng/mL (*p* = 0.031) and 5.9 ng/mL (*p* = 0.008), respectively. 

Participants involved in cleaning volcanic ash showed higher levels of inorganic elements compared to those who did not engage in cleaning activities, with a notable difference in Ni levels. Active cleaners showed a median Ni concentration of 3.9 ng/mL, compared to 2.1 ng/mL in non-cleaners (*p* = 0.031). However, individuals who did not participate in cleaning had higher levels of Sr, with a median concentration of 15.9 ng/mL (*p* = 0.0158). Daily cleaning activities were also associated with marginally elevated levels of certain elements. For instance, daily cleaners recorded a median Cu concentration of 874.3 ng/mL and a Cs concentration of 1.8 ng/mL, whereas those cleaning only once a month had lower concentrations, with Cu at 769.25 ng/mL (*p* = 0.003) and Cs at 1.5 ng/mL (*p* = 0.042). Additionally, spending more than five hours daily in outdoor environments was significantly associated with increased Se (median 112.7 ng/mL) and Pb (median 6.6 ng/mL) levels, while Cu levels were lower (median 826.9 ng/mL), as detailed in Table 4 (Supplementary Table S4).

### 3.5. Correlation Between Main Sociodemographic Factors and Level of Volcano Exposure with Concentration of Inorganic Elements 

**Figure 2:**
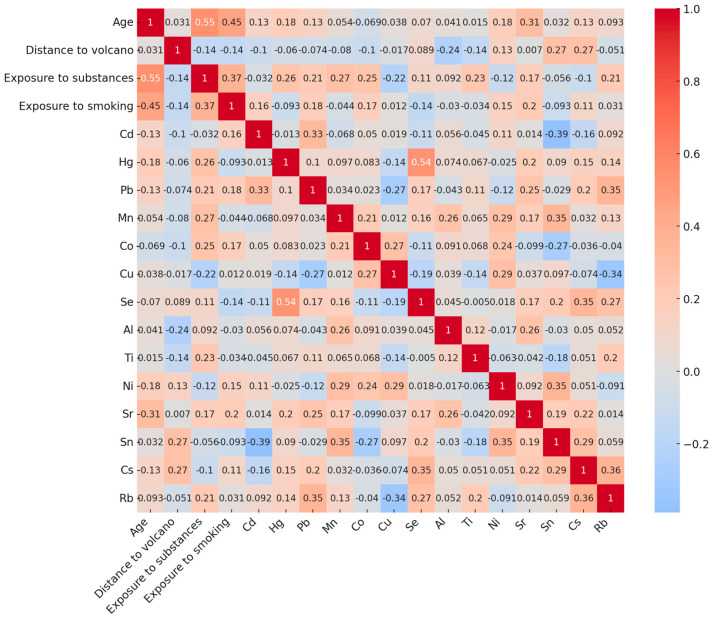
Heatmap plot representing the correlation matrix between numerical sociodemographic variables, types of exposure, and inorganic element concentrations relevant to the study. Positive values (red) indicate a positive relationship between two variables, while negative values (blue) signify a negative relationship.

Spearman’s correlation matrix revealed strong correlations between participant age, years of smoking, and years of occupational exposure to toxic substances. Additionally, a robust correlation was observed between Se and Hg (*p* < 0.001). Furthermore, proximity to the volcano was significantly associated with higher levels of Al (*p* = 0.009) and Ti (*p* = 0.021) (Figure 2).

## 4. Discussion

The results presented in this study are based on the analysis of participants residing in the western region of the ISVOLCAN cohort. In this research, we quantified inorganic contaminants in 393 individuals from the area of La Palma most impacted by the Tajogaite volcano eruption. The results indicate that 14 inorganic elements were detected in the blood samples of more than one-third of the studied population.

It is known that volcanic emissions may represent significant health risks for populations living near active volcanoes, from psychological effects to acute respiratory diseases, and ocular infections [2,6,13,32]. In our study, the main inorganic pollutants detected in over 30% of blood samples were Cd, Hg, Pb, Mn, Co, Cu, Se, Al, Ti, Ni, Sr, Sn, Cs, and Rb. The relatively high detection percentages of Cu, Se, Mn, and Co and given that the levels measured are of the same order of magnitude as the average data reported in the literature for human blood (biological variability) might reflect normal physiological requirements for metabolic processes [33,34,35]. In addition, significant correlations were found between Cd and Pb, Cu and Se, and Rb with both Cu and Se. Similar correlations were found in scalp hair samples of men chronically exposed to volcanogenic metals in the Azores [36]. Nonetheless, current knowledge does not allow for the establishment of definitive thresholds for what constitutes normal concentrations of these elements in our blood samples [37]. Consequently, their application in risk assessment remains unfeasible at this time, despite their concentrations aligning below the upper limit values listed by ATSDR [38] and Human biomonitoring (HBM) Commission [39]. 

Additionally, this article evaluates the presence of rare earth elements. Although the only elements that exceeded 15% in prevalence were yttrium (17%) and cerium (15%), these elements have been detected in other studies related to rare earth elements, but primarily in mining regions [40]. Furthermore, studies on the general adult population in Spain have reported detection rates exceeding 20% for Eu, Tm, In, and Ru [41]. To date, no studies have reported the presence of these elements in human blood samples from individuals exposed to volcanic eruptions. The detection of these elements in our study provides a valuable reference point for conducting further research and highlights the importance of ongoing biomonitoring in populations exposed to volcanic emissions.

Regarding the differences between sociodemographic characteristics and the levels of frequent inorganic contaminants, our study found significant differences. Men exhibited higher concentrations of Pb and Rb, while women showed higher levels of Cu, Al, and Ni. These differences can be attributed to a combination of factors. Occupational exposure plays a role, as men are more often employed in industries with higher lead exposure, such as construction and mining [42]. Physiological factors, including hormonal influences, also contribute; women’s higher estrogen levels can affect copper absorption and distribution. Additionally, genetic, epigenetic, and behavioral factors, such as differences in diet, use of cosmetics, body mass, and composition, further explain these disparities [43,44,45]. Apart from this, participants over the age of 50 showed significantly higher concentrations of Hg and Sr. This can be attributed to accumulation over time, historical environmental exposures, such as consuming contaminated fish, dental amalgams, possible occupational exposures [46,47,48], and changes in elimination capacity due to age-related renal and hepatic function changes and bone remodeling. Older individuals may accumulate more Sr in their bones due to prolonged exposure through diet and drinking water [49,50]. These factors could explain why older individuals tend to have higher levels of these heavy metals compared to younger individuals.

In relation to the distance to volcano, the western population living near the volcano exhibited significantly elevated concentrations of Al, Ti, and Sn, with these elements present in more than one-third of the studied population. This can be attributed to the volcanic emissions containing significant amount of these elements, which can be mobilized in the environment, and could lead to population intake through direct inhalation or oral ingestion, or drinking of contaminated water or consumption of contaminated food [10,51]. In contrast, Rb was found in 100% of the population, regardless of the distance from the volcano. This aligns with a study on White Island, New Zealand, where high excretion levels of Al and Rb were observed post-acute exposure to fumaroles. These elements were suggested as potential biomarkers of volcanic emission exposure [52]. A study on Volcán de Fuego in Guatemala found increased urinary concentrations of As, Cd, and Pb in workers close to the volcano, while high Ni levels were only found in those from more distant highland areas [53]. In our study, no significant differences were found in the concentrations of Cd, Hg, Pb, Mn, Co, Cu, Ni, Sr, Cs, and Rb based on proximity to the volcano, likely due to the small size of the island and uniform exposure levels in our sample population. The concentrations of the same elements in the population of the eastern region, which was less affected by the eruption, will be analyzed in future studies. Comparing the results between the two regions will provide valuable insights.

These inorganic toxicants may originate from the release of gases and volcanic particles during eruptions, but might also reflect the geological background and/or result from anthropogenic pollution. A study analyzing the chemical composition of the water leachates of ash from the Tajogaite volcano predicted Al concentrations in open-water supply catchments exceeding potability values for humans, although the study’s analysis deemed these levels relatively non-toxic but capable of altering organoleptic properties, reducing water acceptability as a beverage [54]. This finding may account for the elevated Al levels detected in one-third of our study population compared to ATSDR reference values. The ATSDR has documented that exposure to high levels of Al can lead to neurological, respiratory, and skeletal effects [38]. Ruggieri et al. (2023) reported that barium levels in areas near the volcano exceeded WHO human consumption limits in only two ash samples [54]. Although there are no established reference levels for barium in human blood, the WHO sets an acceptable consumption limit of 1.3 mg/L [55]. In our study, barium was detected in 22% of the participants. Given that its intake is strictly regulated by international agencies, barium should be closely monitored in biomonitoring efforts.

Among the elements found in Tajogaite ash, fluoride has significant toxicological importance [54]. This element has the potential to deposit in soil and water, entering the food chain through plants and animals [56,57]. Although our study did not assess fluoride, we found elevated Al and Ti levels in blood of participants residing less than 6.5 km from the volcano. A study on banana cultivation in La Palma post-eruption also found high concentrations of Al, Ti, and Pb in banana flesh, indicating their entry into the food chain [58]. However, the presence of inorganic elements in the environment does not necessarily imply direct or proportional incorporation into the human body, as this process is influenced by the bioaccessibility and the bioavailability of each element [59]. Therefore, this underscores the importance of biomonitoring these contaminants to assess exposure and guide public health interventions.

Other sources of exposure to these persistent pollutants may be those derived from occupational exposure. Although only 18% of participants reported occupational exposure to toxic substances, those with over 15 years of exposure exhibited significantly higher concentrations of Hg and Mn compared to those with shorter exposure periods. This can be attributed to bioaccumulation; for example, mercury has a long half-life in the human body, and chronic occupational exposure allows gradual accumulation due to its slow elimination rate [47]. Jobs involving inhalation of manganese dust and fumes, such as mining and steel production, expose workers to high levels of manganese, resulting in significant accumulations over time [60,61]. Participants with less than 15 years of occupational exposure to toxic substances showed lower median levels of several elements, indicating a dose–response relationship between exposure duration and inorganic pollutant accumulation.

Concerning the exposure level of the population during the eruption, no significant differences were observed. However, individuals who reported spending less time outdoors had significantly lower Pb and Se levels compared to those who spent more hours outside. One might expect these lower concentrations to be associated with the use of protective masks, but no significant correlations were found with mask usage or the use of high particle projection tools. Despite evidence suggesting that protective measures like mask use and limiting outdoor activities are effective in mitigating health risks [62], our data emphasize the importance of proximity to the eruption zone and the activities undertaken during and after the eruption in influencing the presence of inorganic pollutants in the human body.

In this study, smoking exposure, smoker status, and the duration of smoking exposure were associated with higher concentrations of Cd and Pb. Similar findings have been reported in a study assessing the influence of smoking habits on cadmium and lead blood levels in the Serbian adult population [63], as well as in the BIOAMBIENT.ES project, which found that smoking increases cadmium levels in urine [64]. It is known that tobacco contains various levels of inorganic elements such as Al, As, Ba, Ca, Cd, Co, Cr, Cu, Fe, K, Na, Ni, Rb, Si, Sr, and Zn, which can vary between different commercial brands [65,66]. Galażyn-Sidorczuk et al. (2008) found that tobacco is a significant source of cadmium because tobacco plants absorb cadmium and lead from the soil and accumulate them in their leaves. During combustion, these elements are inhaled directly, increasing their bioaccumulation in smokers, particularly in the kidneys and liver, serving as biomarkers of renal damage compared to non-smokers [67,68]. 

### Limitations and Strengths

This study serves as the baseline assessment of the ISVOLCAN cohort, and as such, its main limitations include the inability to track changes over time or establish causality. The focus on a specific geographic area may limit the generalization of the results to other volcanic or environmental conditions. Additionally, reliance on self-reported data might introduce bias. The analysis was also limited to a specific group of trace elements in a single type of sample, potentially underestimating the full spectrum of exposure and associated health risks. Moreover, some relevant volcanic pollutants—such as fluoride, a known toxic component of volcanic emissions—were not included in the analysis, representing a limitation in the characterization of total exposure.

Despite these limitations, our study was conducted using blood samples, which provide reliable data on acute exposure, allowing for comparative analysis with established ATSDR thresholds and other studies. Whole blood is widely recognized as the most suitable specimen for measuring inorganic contaminant levels, as it accurately reflects the bioavailability of the contaminant and, consequently, its potential toxic effects. This method offers significant advantages over other biological samples, such as urine or hair. Furthermore, ISVOLCAN is designed as a 10-year longitudinal cohort study, which will allow future follow-up to evaluate medium- and long-term health effects related to volcanic exposure in a large sample of the adult general population residing on La Palma.

## 5. Conclusions

To our knowledge, this is the first article on biomonitoring in blood samples from a general population exposed to a volcanic eruption. Our results indicate that proximity to the volcano resulted in elevated levels of Al and Ti, frequent ash cleaning was linked to higher Cu and Ni levels, and spending more hours outdoors was associated with higher level of Pb and Se. Notably, significant concentrations of Al and Ti suggest their potential as exposure biomarkers. Further research is essential to understand the long-term health implications of chronic exposure to these inorganic elements through various routes of entry into the body, which will guide future public health strategies and interventions. Implementing such measures can significantly mitigate the health impact of volcanic eruptions and enhance community resilience against environmental hazards.

## Figures and Tables

**Table 1:** Sociodemographic characteristics of participants from the western region of La Palma.

Variables		Total n = 393
		n (%)
**Age category (years)**	≤50	187 (47.58)
	>50	206 (52.42)
**Sex**	Male	170 (43.26)
	Female	223 (56.74)
**Distance (usual residence) to volcano (km) median (IQR)**		6.46 (4.64–7.12)
**Distance (usual residence) to volcano (km)**	<6.5	201 (51.15)
	≥6.5	192 (48.85)
**Employment status**	Active	238 (60.6)
	Inactive	155 (39.4)
**Occupational exposure to toxic substances**	No	323 (82.19)
	Yes	70 (17.81)
**Years of occupational exposure to toxic substances; median (IQR) (n = 58)**		15.5 (5–23)
**Years of occupational exposure to toxic substances (n = 58)**	<15 years	25 (43.10)
	≥15 years	33 (56.90)
**Smoking exposure**	No	220 (55.98)
	Yes	173 (44.02)
**Years of exposure to smoking; median (IQR) (n = 168)**		20 (10-30)
**Years of exposure to smoking (n = 168)**	<20 years	65 (38.69)
	≥20 years	103 (61.31)
**Smoker status**	Current smoker	78 (19.85)
	Ex-smoker	95 (24.17)
	Never smoked	220 (55.98)
**Volcanic ash cleaning**	No	32 (8.14)
	Yes	361 (91.86)
**Cleaning tools (n = 361)**	High (particle projection)	333 (92.24)
	Moderate (particle projection)	21 (5.82)
	Low (particle projection)	7 (1.94)
**Cleaning frequency (n = 356)**	≥1 once a day	186 (52.25)
	1–6 times per week	155 (43.54)
	Every 15 days/monthly	15 (4.21)
**Daily hours spent in outdoor environments**	No or <1 h	41 (10.43)
	1–5 h	150 (38.17)
	>5 h	202 (51.4)

**Abbreviations**: IQR: interquartile range; ND: no data. Note: When the number of observations differs from the total sample size, the exact count used for percentage calculations is indicated alongside the variable. The complete table with all of the studied variables is available in Supplementary Table S1.

**Table 2:** Summary of quantitative levels of inorganic elements in whole blood (ng/mL) among participants from the western region (n = 393).

Inorganic Elements Included in the ATSDR’s Priority Pollutant List (2022)	Frequency of Detection (%)	Mean (SD)	Median (IQR)	95% Confidence Interval (Lower Limit-Upper Limit)
**Al (Aluminium)**	30.53	518.6 (949.59)	80.36 (39.42–180.76)	(346.95–690.24)
**Cd (Cadmium)**	45.80	0.46 (0.69)	0.23 (0.12–0.45)	(0.36–0.56)
**Co (Cobalt)**	44.02	0.26 (0.24)	0.21 (0.13–0.29)	(0.23–0.30)
**Cs (Cesium)**	95.42	1.77 (0.86)	1.66 (1.14–2.32)	(1.69–1.86)
**Cu (Copper)**	100.00	888.33 (189.32)	842.33 (777.62–954.77)	(869.56–907.11)
**Hg (Mercury)**	98.73	3.87 (3.15)	3.13 (1.58–5.1)	(3.55–4.18)
**Mn (Manganese)**	96.44	7.87 (2.88)	7.48 (6.12–9.48)	(7.58–8.16)
**Ni (Nickel)**	40.46	10.96 (62.64)	3.86 (1.9–5.58)	(1.15–20.78)
**Pb (Lead)**	92.11	7.73 (7.18)	6.04 (3.65–9.75)	(6.99–8.47)
**Se (Selenium)**	100.00	112.77 (30.04)	109.24 (94.06–126.07)	(109.79–115.75)
**Sr (Strontium)**	98.47	14.24 (6.08)	13.6 (10.48–17.05)	(13.63–14.85)
**Other inorganic elements not included in the priority pollutant list**				
**Rb (Rubidium)**	100.00	1869.86 (381.97)	1848.83 (1621.31–2088.65)	(1831.98–1907.75)
**Sn (Tin)**	32.57	6.84 (5.25)	4.76 (2.87–10.28)	(5.92–7.76)
**Ti (Titanium)**	69.72	8.26 (4.18)	7.22 (5.41–10.32)	(7.77–8.76)

**Abbreviations**: IQR: interquartile range; LOQ: limit of quantification; SD: standard deviation. **Note**: This is a shortened version presented due to space limitations. Full data for all inorganic elements are provided in Supplementary Table S2.

**Table 3:** Summary of quantitative levels of inorganic pollutants (ng/mL) in whole blood by sociodemographic characteristics among participants from the western region.

	Inorganic Elements	Cd	Hg	Pb	Mn	Cu	Al	Ni	Sr
	% of Detection	46%	99%	92%	96%	100%	31%	41%	99%
**Variables**		Median (IQR)	Median (IQR)	Median (IQR)	Median (IQR)	Median (IQR)	Median (IQR)	Median (IQR)	Median (IQR)
**Age category (years)**	≤50	0.18 (0.11–0.34)	2.52 (1.49–4.29) **	5.79 (3.21–9.43)	7.37 (5.82–9.65)	830.95 (769.41–936.97)	81.04 (37.42–189.18)	3.40 (1.84–5.10)	12.50 (9.51–15.26) ***
	>50	0.25 (0.12–0.51)	3.60 (1.76–5.87)	6.42 (4.09–9.78)	7.53 (6.16–9.36)	855.81 (783.72–963.35)	79.67 (43.33–163.65)	4.34 (2.03–6.25)	14.89 (12.02–18.80)
**Sex**	Male	0.21 (0.12–0.47)	3.58 (1.59–5.86)	8.72 (5.09–13.11) ***	7.52 (6.19–9.27)	793.91 (740.04–852.86) ***	57.28 (32.81–169.82) *	2.68 (1.66–4.72) **	13.55 (10.53–16.35)
	Female	0.23 (0.12–0.44)	2.89 (1.57–4.75)	4.70 (3.01–7.23)	7.37 (5.83–10.10)	913.97 (820.57–1021.53)	100.07 (55.46–273.15)	4.40 (2.31–6.56)	13.84 (10.43–17.66)
**Years of occupational exposure to toxic substances**	<15 years	0.33 (0.12–0.44)	1.88 (1.21–2.69) *	6.60 (3.39–9.39)	6.85 (5.95–9.27) *	849.24 (794.79–957.80) *	84.80 (52.92–243.65)	4.02 (2.18–4.27)	13.76 (11.09–17.91)
	≥15 years	0.21 (0.13–0.50)	3.98 (2.00–6.32)	8.79 (5.38–14.13)	8.55 (6.82–10.48)	790.40 (711.20–838.32)	100.49 (28.06–2.046.19)	2.16 (1.56–4.72)	13.88 (10.32–18.23)
**Smoking exposure**	No	0.14 (0.10–0.25) ***	3.12 (1.54–5.21)	4.99 (3.16–8.17) ***	7.65 (6.28–9.93) *	854.02 (778.01–972.25)	79.67 (40.08–123.26)	3.89 (1.93–5.85)	13.22 (10.37–16.28)
	Yes	0.34 (0.19–0.78)	3.13 (1.60–4.94)	7.57 (4.70–12.32)	7.34 (5.81–9.06)	831.25 (774.12–940.72)	92.97 (37.10–1.329.38)	3.83 (1.84–5.39)	13.98 (10.82–17.72)
**Smoker status**	Current smoker	0.55 (0.29–1.04) ***	2.51 (1.46–4.33)	7.54 (4.72–13.25) ***	6.77 (5.81–8.77)	818.37 (764.52–929.48)	96.52 (37.10–1.885.53)	3.25 (1.84–6.08)	13.86 (10.53–17.37)
	Ex-smoker	0.22 (0.11–0.34)	3.69 (1.96–5.63)	7.62 (4.70–11.32)	7.44 (5.85–9.31)	842.33 (779.72–960.00)	65.13 (38.71–732.49)	3.91 (2.21–5.08)	14.12 (11.34–18.45)
	Never smoked	0.14 (0.10–0.25)	3.12 (1.54–5.21)	4.99 (3.16–8.17)	7.65 (6.28–9.93)	854.02 (778.01–972.25)	79.67 (40.08–123.26)	3.89 (1.93–5.85)	13.22 (10.37–16.28)

**Abbreviations**: ***p* value significance levels**: *: <0.05; **: <0.01; ***: <0.001. **Statistical analysis**: Mann–Whitney U test for dichotomous variables; Kruskal–Wallis test for polytomous variables. **Note**: A condensed version is shown here due to space constraints. Full results for all 14 inorganic elements and variables are available in Supplementary Table S3.

**Table 4:** Summary of quantitative levels of inorganic elements (ng/mL) in whole blood of the participants from the western region in relation to the level of exposure to Tajogaite volcano during eruption.

	Inorganic Elements	Cu	Se	Al	Ti	Ni	Sr	Sn	Cs
	% of Detection	100%	100%	31%	70%	41%	99%	33%	95%
**Variables**		Median (IQR)	Median (IQR)	Median (IQR)	Median (IQR)	Median (IQR)	Median (IQR)	Median (IQR)	Median (IQR)
**Distance (usual residence) to volcano (km)**	<6.5	845.46 (781.52–946.44)	107.93 (90.47–125.51) *	107.82 (49.45–323.00) *	7.60 (5.70–11.40) *	3.68 (1.82–5.24)	13.76 (9.98–16.96)	4.37 (2.39–7.00) **	1.54 (1.06–2.24)
	≥6.5	839.18 (771.31–966.81)	111.51 (96.57–129.42)	55.33 (36.81–100.41)	6.88 (5.04–9.36)	4.02 (2.38–6.56)	13.47 (10.69–17.21)	5.85 (3.63–12.22)	1.73 (1.23–2.33)
**Volcanic ash cleaning**	No	809.56 (777.16–896.52)	107.38 (92.52–122.81)	103.61 (30.62–2295.76)	7.05 (5.80–11.06)	2.09 (1.34–2.77) *	15.86 (12.53–18.90) *	3.58 (1.93–4.60)	1.54 (0.97–2.64)
	Yes	845.23 (777.62–960.00)	109.36 (94.26–126.20)	79.67 (40.08–169.82)	7.24 (5.39–10.32)	3.93 (1.98–5.70)	13.55 (10.32–16.64)	4.93 (2.89–10.82)	1.67 (1.16–2.24)
***Cleaning frequency***	> = 1 once a day	874.27 (788.01–1006.37) **	111.19 (92.64–132.13)	83.49 (41.60–189.18)	6.98 (5.28–9.75)	4.18 (2.14–6.68)	13.57 (10.27–16.28)	5.13 (3.04–11.71)	1.77 (1.32–2.37) *
	1–6 times per week	834.38 (769.41–906.15)	109.58 (96.64–123.37)	79.70 (38.71–163.87)	7.28 (5.36–10.29)	3.93 (1.98–5.14)	13.56 (10.49–16.67)	4.55 (2.71–7.52)	1.55 (1.05–2.15)
	Every 15 days/monthly	769.25 (740.04–932.50)	103.78 (95.33–127.23)	62.49 (52.68–116.39)	11.66 (8.01–12.95)	2.83 (1.47–4.19)	11.11 (7.13–14.45)	3.98 (2.86–11.18)	1.52 (0.82–2.32)
**Daily hours spent in outdoor environments**	None or <1 h	883.61 (820.59–987.01) **	99.28 (80.12–115.56) **	93.48 (46.54–172.33)	6.48 (4.62–9.47)	3.91 (1.98–6.07)	13.24 (10.25–16.96)	5.10 (2.60–10.24)	1.50 (1.21–2.29)
	1–5 h	866.66 (783.96–973.63)	108.92 (91.50–126.94)	88.78 (39.05–169.82)	8.23 (5.85–11.77)	4.29 (1.90–6.08)	14.10 (10.50–18.23)	4.57 (2.41–7.33)	1.74 (1.15–2.33)
	>5 h	826.93 (770.53–916.70)	112.69 (97.52–127.07)	73.04 (39.25–281.02)	7.05 (5.17–9.48)	3.46 (1.90–5.09)	13.47 (10.49–16.17)	4.97 (3.15–11.07)	1.61 (1.09–2.37)

**Abbreviations**: ***p* value significance levels**: *: <0.05; **: <0.01. **Statistical tests**: Mann–Whitney U test for dichotomous variables; Kruskal–Wallis test for polytomous variables. **Note**: Due to space limitations, this table presents a condensed version of the results. The full dataset, including detailed information for all 14 inorganic elements and associated variables, is provided in Supplementary Table S4.

## Data Availability

Data will be made available on request due to sensitive or confidential information of participants.

## Supplementary Materials

The following supporting information can be downloaded at: https://www.mdpi.com/article/10.3390/toxics13070581/s1; Data S1: The LOD and LOQ values for each analyte, along with precision and accuracy data from CRM and spiked samples; Table S1: Sociodemographic characteristics of participants from the western region of La Palma; Table S2: Quantitative levels of inorganic elements in whole blood (ng/mL) among participants from the western region; Table S3: Quantitative levels of inorganic pollutants (ng/mL) in whole blood by sociodemographic characteristics among participants from the western region; Table S4: Quantitative levels of Trace elements (ng/mL) in whole blood of the participants from the western region in relation to the level of exposure to Tajogaite volcano during eruption.

## Abbreviations

The following abbreviations are used in this manuscript:

ATSDR	Agency for Toxic Substances and Disease Registry
HF	Hydrogen Fluoride
HCl	Hydrogen Chloride
H_2_S	Hydrogen Sulfide
ICP-MS	Inductively Coupled Plasma Mass Spectrometry
SO_2_	Sulfur Dioxide
WHO	World Health Organization

## References

[1] Gudmundsson G., Larsen G. (2016). Effects of volcanic eruptions on human health in Iceland. Review. Laeknabladid.

[2] Stewart C., Damby D.E., Horwell C.J., Elias T., Ilyinskaya E., Tomašek I., Longo B.M., Schmidt A., Carlsen H.K., Mason E. (2022). Volcanic Air Pollution and Human Health: Recent Advances and Future Directions. Bull. Volcanol..

[3] Horwell C.J., Baxter P.J. (2006). The Respiratory Health Hazards of Volcanic Ash: A Review for Volcanic Risk Mitigation. Bull. Volcanol..

[4] Gudmundsson G. (2011). Respiratory Health Effects of Volcanic Ash with Special Reference to Iceland. A Review. Clin. Respir. J..

[5] Lombardo D., Ciancio N., Campisi R., Di Maria A., Bivona L., Poletti V., Mistretta A., Biggeri A., Di Maria G. (2013). A Retrospective Study on Acute Health Effects Due to Volcanic Ash Exposure during the Eruption of Mount Etna (Sicily) in 2002. Multidiscip. Respir. Med..

[6] Forbes L., Jarvis D., Potts J., Baxter P.J. (2003). Volcanic Ash and Respiratory Symptoms in Children on the Island of Montserrat, British West Indies. Occup. Environ. Med..

[7] Eychenne J., Gurioli L., Damby D., Belville C., Schiavi F., Marceau G., Szczepaniak C., Blavignac C., Laumonier M., Gardés E. (2022). Spatial Distribution and Physicochemical Properties of Respirable Volcanic Ash from the 16-17 August 2006 Tungurahua Eruption (Ecuador), and Alveolar Epithelium Response In-Vitro. Geohealth.

[8] Damby D.E., Horwell C.J., Baxter P.J., Kueppers U., Schnurr M., Dingwell D.B., Duewell P. (2018). Volcanic Ash Activates the NLRP3 Inflammasome in Murine and Human Macrophages. Front. Immunol..

[9] Sauzéat L., Eychenne J., Gurioli L., Boyet M., Jessop D.E., Moretti R., Monrose M., Holota H., Beaudoin C., Volle D.H. (2022). Metallome Deregulation and Health-Related Impacts Due to Long-Term Exposure to Recent Volcanic Ash Deposits: New Chemical and Isotopic Insights. Sci. Total Environ..

[10] Ilyinskaya E., Mason E., Wieser P.E., Holland L., Liu E.J., Mather T.A., Edmonds M., Whitty R.C.W., Elias T., Nadeau P.A. (2021). Rapid Metal Pollutant Deposition from the Volcanic Plume of Kīlauea, Hawai’i. Commun. Earth Environ..

[11] Calkins J., Delmelle P. (2021). Quantitative Analysis of Persistent Volcanic Fluoride Risk Reveals Differential Exposure Pathways for Adults and Children Downwind of Masaya Volcano, Nicaragua. Bull. Volcanol..

[12] Hansell A., Oppenheimer C. (2004). Health Hazards from Volcanic Gases: A Systematic Literature Review. Arch. Environ. Health Int. J..

[13] Carlsen H.K., Ilyinskaya E., Baxter P.J., Schmidt A., Thorsteinsson T., Pfeffer M.A., Barsotti S., Dominici F., Finnbjornsdottir R.G., Jóhannsson T. (2021). Increased Respiratory Morbidity Associated with Exposure to a Mature Volcanic Plume from a Large Icelandic Fissure Eruption. Nat. Commun..

[14] Kochi T., Iwasawa S., Nakano M., Tsuboi T., Tanaka S., Kitamura H., Wilson D.J., Takebayashi T., Omae K. (2017). Influence of Sulfur Dioxide on the Respiratory System of Miyakejima Adult Residents 6 Years after Returning to the Island. J. Occup. Health.

[15] Lamela P.A., Navoni J.A., Pérez R.D., Pérez C.A., Vodopivez C.L., Curtosi A., Bongiovanni G.A. (2019). Analysis of Occurrence, Bioaccumulation and Molecular Targets of Arsenic and Other Selected Volcanic Elements in Argentinean Patagonia and Antarctic Ecosystems. Sci. Total. Environ..

[16] Malandrino P., Russo M., Ronchi A., Minoia C., Cataldo D., Regalbuto C., Giordano C., Attard M., Squatrito S., Trimarchi F. (2016). Increased Thyroid Cancer Incidence in a Basaltic Volcanic Area Is Associated with Non-Anthropogenic Pollution and Biocontamination. Endocrine.

[17] Centers for Disease Control and Prevention (CDC) The ATSDR 2022 Substance Priority List. https://www.atsdr.cdc.gov/programs/substance-priority-list.html.

[18] Vigneri R., Malandrino P., Gianì F., Russo M., Vigneri P. (2017). Heavy Metals in the Volcanic Environment and Thyroid Cancer. Mol. Cell. Endocrinol..

[19] Milford C., Torres C., Vilches J., Gossman A.-K., Weis F., Suárez-Molina D., García O.E., Prats N., Barreto Á., García R.D. (2023). Impact of the 2021 La Palma Volcanic Eruption on Air Quality: Insights from a Multidisciplinary Approach. Sci. Total Environ..

[20] Ruano-Ravina A., Acosta O., Díaz Pérez D., Casanova C., Velasco V., Peces-Barba G., Barreiro E., Cañas A., Castaño A., Cruz Carmona M.J. (2023). A Longitudinal and Multidesign Epidemiological Study to Analyze the Effect of the Volcanic Eruption of Tajogaite Volcano (La Palma, Canary Islands). ASHES Study Protocol. Environ. Res..

[21] Gobierno de Canarias Isla de La Palma. https://www3.gobiernodecanarias.org/medusa/wiki/index.php?title=Isla_de_La_Palma.

[22] Rodríguez-Pérez M.C., Ferrer M.E.F., Boada L.D., Pérez A.D.A., Aguilar M.C.D., Jerónimo J.F.F., Talavera I.G., Gangotena L.V., de la Torre A.H., Simbaña-Rivera K. (2024). Health Impact of the Tajogaite Volcano Eruption in La Palma Population (ISVOLCAN Study): Rationale, Design, and Preliminary Results from the First 1002 Participants. Environ. Health.

[23] Rubio-Armendáriz C., Paz S., Gutiérrez Á.J., González-Weller D., Revert C., Hardisson A. (2021). Human Exposure to Toxic Metals (Al, Cd, Cr, Ni, Pb, Sr) from the Consumption of Cereals in Canary Islands. Foods.

[24] von Suchodoletz H., Glaser B., Thrippleton T., Broder T., Zang U., Eigenmann R., Kopp B., Reichert M., Ludwig Z. (2013). The Influence of Saharan Dust Deposits on La Palma Soil Properties (Canary Islands, Spain). Catena.

[25] Dóniz-Páez J., Németh K., Becerra-Ramírez R., Hernández W., Gosálvez R.U., Escobar E., González E. (2022). Tajogaite 2021 Eruption (La Palma, Canary Islands, Spain): An Exceptional Volcanic Heritage to Develop Geotourism. Proceedings.

[26] Instituto Geográfico Nacional La Palma (Isla) (1996). Mapas Topográficos. https://www.ign.es/web/catalogo-cartoteca/resources/html/017034.html.

[27] (2021). PEVOLCA Actualización de la actividad volcánica en Cumbre Vieja (La Palma); Canarias. https://info.igme.es/eventos/erupcion-volcanica-la-palma/pevolca.

[28] Gobierno de Canarias Volcán de Tajogaite o Cabezavaca. https://www3.gobiernodecanarias.org/medusa/wiki/index.php?title=Volc%C3%A1n_de_Tajogaite_o_Cabezavaca.

[29] European Commission Elementary Occupations. https://esco.ec.europa.eu/en/classification/occupation.

[30] Agency for Toxic Substances and Disease Registry Módulo I—Introducción a la Toxicología. https://www.atsdr.cdc.gov/es/training/toxicology_curriculum/modules/1/es_module1.html.

[31] Henríquez-Hernández L.A., Zumbado M., Rodríguez-Hernández Á., Duarte-Lopes E., Lopes-Ribeiro A.L., Alfama P.M., Livramento M., Díaz-Díaz R., Bernal-Suárez M.D.M., Boada L.D. (2023). Human Biomonitoring of Inorganic Elements in a Representative Sample of the General Population from Cape Verde: Results from the PERVEMAC-II Study. Chemosphere.

[32] Baxter P.J., Ing R., Falk H., French J., Stein G.F., Bernstein R.S., Merchant J.A., Allard J. (1981). Mount St Helens Eruptions, May 18 to June 12, 1980. An Overview of the Acute Health Impact. JAMA.

[33] Gaetke L.M., Chow-Johnson H.S., Chow C.K. (2014). Copper: Toxicological Relevance and Mechanisms. Arch. Toxicol..

[34] Genchi G., Lauria G., Catalano A., Sinicropi M.S., Carocci A. (2023). Biological Activity of Selenium and Its Impact on Human Health. Int. J. Mol. Sci..

[35] Czarnek K., Terpiłowska S., Siwicki A.K. (2015). Selected Aspects of the Action of Cobalt Ions in the Human Body. Cent. Eur. J. Immunol..

[36] Amaral A.F.S., Arruda M., Cabral S., Rodrigues A.S. (2008). Essential and Non-Essential Trace Metals in Scalp Hair of Men Chronically Exposed to Volcanogenic Metals in the Azores, Portugal. Environ. Int..

[37] Bevan R., Levy L. (2024). Biomonitoring for Workplace Exposure to Copper and Its Compounds Is Currently Not Interpretable. Int. J. Hyg. Environ. Health.

[38] Agency for Toxic Substances and Disease Registry ToxGuides^TM^|ATSDR. https://wwwn.cdc.gov/TSP/ToxGuides/ToxGuidesLanding.aspx.

[39] Human Biomonitoring (HBM) Commission Reference and Human Biomonitoring (HBM) Values. https://www.umweltbundesamt.de/en/topics/health/commissions-working-groups/human-biomonitoring-commission/reference-hbm-values.

[40] Belisheva N.K., Drogobuzhskaya S.V. (2024). Rare Earth Element Content in Hair Samples of Children Living in the Vicinity of the Kola Peninsula Mining Site and Nervous System Diseases. Biology.

[41] Gasull M., Camargo J., Pumarega J., Henríquez-Hernández L.A., Campi L., Zumbado M., Contreras-Llanes M., Oliveras L., González-Marín P., Luzardo O.P. (2024). Blood Concentrations of Metals, Essential Trace Elements, Rare Earth Elements and Other Chemicals in the General Adult Population of Barcelona: Distribution and Associated Sociodemographic Factors. Sci. Total Environ..

[42] Cañas A.I., Cervantes-Amat M., Esteban M., Ruiz-Moraga M., Pérez-Gómez B., Mayor J., Castaño A., BIOAMBIENT.ES (2014). Blood Lead Levels in a Representative Sample of the Spanish Adult Population: The BIOAMBIENT.ES Project. Int. J. Hyg. Environ. Health.

[43] Huang C.-H., Wang C.-W., Chen H.-C., Tu H.-P., Chen S.-C., Hung C.-H., Kuo C.-H. (2022). Gender Difference in the Associations among Heavy Metals with Red Blood Cell Hemogram. Int. J. Environ. Res. Public Health.

[44] Gade M., Comfort N., Re D.B. (2021). Sex-Specific Neurotoxic Effects of Heavy Metal Pollutants: Epidemiological, Experimental Evidence and Candidate Mechanisms. Environ. Res..

[45] Mendiola J., Moreno J.M., Roca M., Vergara-Juárez N., Martínez-García M.J., García-Sánchez A., Elvira-Rendueles B., Moreno-Grau S., López-Espín J.J., Ten J. (2011). Relationships between Heavy Metal Concentrations in Three Different Body Fluids and Male Reproductive Parameters: A Pilot Study. Environ. Health.

[46] Castaño A., Pedraza-Díaz S., Cañas A.I., Pérez-Gómez B., Ramos J.J., Bartolomé M., Pärt P., Soto E.P., Motas M., Navarro C. (2019). Mercury Levels in Blood, Urine and Hair in a Nation-Wide Sample of Spanish Adults. Sci. Total. Environ..

[47] Mortensen M.E., Caudill S.P., Caldwell K.L., Ward C.D., Jones R.L. (2014). Total and Methyl Mercury in Whole Blood Measured for the First Time in the US Population: NHANES 2011–2012. Environ. Res..

[48] Weiner J.A., Nylander M. (1993). The Relationship between Mercury Concentration in Human Organs and Different Predictor Variables. Sci. Total. Environ..

[49] Shagina N.B., Tolstykh E.I., Zalyapin V.I., Degteva M.O., Kozheurov V.P., Tokareva E.E., Anspaugh L.R., Napier B.A. (2003). Evaluation of Age and Gender Dependences of the Rate of Strontium Elimination 25–45 Years after Intake: Analysis of Data from Residents Living along the Techa River. Rare.

[50] Tolstykh E.I., Degteva M.O., Kozheurov V.P., Shishkina E.A., Romanyukha A.A., Wieser A., Jacob P. (2000). Strontium Metabolism in Teeth and Enamel Dose Assessment: Analysis of the Techa River Data. Radiat. Environ. Biophys.

[51] Stewart C., Johnston D., Leonard G., Horwell C., Thordarson T., Cronin S. (2006). Contamination of Water Supplies by Volcanic Ashfall: A Literature Review and Simple Impact Modelling. J. Volcanol. Geotherm. Res..

[52] Durand M., Florkowski C., George P., Walmsley T., Weinstein P., Cole J. (2004). Elevated Trace Element Output in Urine Following Acute Volcanic Gas Exposure. J. Volcanol. Geotherm. Res..

[53] Neumann N.R., Butler-Dawson J., Krisher L., Jaramillo D., Pilloni D., Waite G., Li Y., Wittels S.B., Schilling K., Newman L.S. (2023). Urinary Concentrations of Metals before and after Volcanic Eruption: A Natural Experiment Surrounding the Eruption of Volcán de Fuego, 2018. Environ. Geochem. Health.

[54] Ruggieri F., Forte G., Bocca B., Casentini B., Bruna Petrangeli A., Salatino A., Gimeno D. (2023). Potentially Harmful Elements Released by Volcanic Ash of the 2021 Tajogaite Eruption (Cumbre Vieja, La Palma Island, Spain): Implications for Human Health. Sci Total Environ..

[55] World Health Organization Guidelines for Drinking-Water Quality: Fourth Edition Incorporating the First and Second Addenda. https://www.who.int/publications/i/item/9789240045064.

[56] Carrera-Beltrán L., Gavilanes-Terán I., Idrovo-Novillo J., Valverde V.H., Rodríguez-Pinos A., Paredes C., Signes-Pastor A.J., Carbonell-Barrachina Á.A. (2024). Environmental Pollution by Heavy Metals within the Area Influenced by the Tungurahua Volcano Eruption—Ecuador. Ecotoxicol. Environ. Saf..

[57] Ferrante M., Fiore M., Ledda C., Cicciù F., Alonzo E., Fallico R., Platania F., Di Mauro R., Valenti L., Sciacca S. (2013). Monitoring of Heavy Metals and Trace Elements in the Air, Fruits and Vegetables and Soil in the Province of Catania (Italy). Ig. E Sanita Pubblica.

[58] Rodríguez-Hernández Á., Díaz-Díaz R., Zumbado M., Bernal-Suárez M.d.M., Acosta-Dacal A., Macías-Montes A., Travieso-Aja M.d.M., Rial-Berriel C., Hernández L.A., Boada L.D. (2022). Impact of Chemical Elements Released by the Volcanic Eruption of La Palma (Canary Islands, Spain) on Banana Agriculture and European Consumers. Chemosphere.

[59] Fernández-Caliani J.C., Giráldez M.I., Barba-Brioso C. (2019). Oral Bioaccessibility and Human Health Risk Assessment of Trace Elements in Agricultural Soils Impacted by Acid Mine Drainage. Chemosphere.

[60] Mauriello M.C., Sbordone C., Montuori P., Alfano R., Triassi M., Iavicoli I., Manno M. (2017). Biomonitoring of Toxic Metals in Incinerator Workers: A Systematic Review. Toxicol. Lett..

[61] Karyakina N.A., Shilnikova N., Farhat N., Ramoju S., Cline B., Momoli F., Mattison D., Jensen N., Terrell R., Krewski D. (2022). Biomarkers for Occupational Manganese Exposure. Crit. Rev. Toxicol..

[62] Steinle S., Sleeuwenhoek A., Mueller W., Horwell C.J., Apsley A., Davis A., Cherrie J.W., Galea K.S. (2018). The Effectiveness of Respiratory Protection Worn by Communities to Protect from Volcanic Ash Inhalation. Part II: Total Inward Leakage Tests. Int. J. Hyg. Environ. Health.

[63] Repić A., Bulat P., Antonijević B., Antunović M., Džudović J., Buha A., Bulat Z. (2020). The Influence of Smoking Habits on Cadmium and Lead Blood Levels in the Serbian Adult People. Environ. Sci Pollut. Res. Int..

[64] López-Herranz A., Cutanda F., Esteban M., Pollán M., Calvo E., Pérez-Gómez B., Victoria Cortes M., Castaño A., BIOAMBIENT.ES (2016). Cadmium Levels in a Representative Sample of the Spanish Adult Population: The BIOAMBIENT.ES Project. J. Expo. Sci. Environ. Epidemiol..

[65] Halstead M.M., Watson C.H., Pappas R.S. (2015). Electron Microscopic Analysis of Surface Inorganic Substances on Oral and Combustible Tobacco Products. J. Anal. Toxicol..

[66] Oliveira H., Fernandes E.A.N., Bacchi M.A., Sarriés G.A., Tagliaferro F.S. (2000). Tobacco Element Composition Determined by INAA. J. Radioanal. Nucl. Chem..

[67] Galażyn-Sidorczuk M., Brzóska M.M., Moniuszko-Jakoniuk J. (2008). Estimation of Polish Cigarettes Contamination with Cadmium and Lead, and Exposure to These Metals via Smoking. Environ. Monit. Assess.

[68] Mortada W.I., Sobh M.A., El-Defrawy M.M. (2004). The Exposure to Cadmium, Lead and Mercury from Smoking and Its Impact on Renal Integrity. Med. Sci. Monit..

