# Supplementary material for: Biomonitoring of Inorganic Pollutants in Blood Samples of Population Affected by the Tajogaite Eruption: The ISVOLCAN Study in Spain
Source: Toxics. 2025 Jul 10;13(7):581. doi: 10.3390/toxics13070581 (PMC12298855; doi:10.3390/toxics13070581)
Supplement: Supplementary file 1 [file toxics-13-00581-s001.zip › Supplementary tables.pdf]

Table S1 Sociodemographic characteristics of participants from the western region of La Palma.

**Variables**		**Total n=393**
		n (%)
**Age (years) median (IQR)**		51 (40-59)
**Age category (years)**	≤50	187 (47.58)
	>50	206 (52.42)
**Gender**	Male	170 (43.26)
	Female	223 (56.74)
**Distance (usual residence) to volcano (km) median (IQR)**		6.46 (4.64-7.12)
**Distance (usual residence) to volcano (km)**	<6.5	201 (51.15)
	≥6.5	192 (48.85)
**Place of residence**	El Paso	119 (30.28)
	Fuencaliente	1 (0.25)
	Los Llanos de Aridane	199 (50.64)
	Puntagorda	10 (2.54)
	Tazacorte	55 (13.99)
	Tijarafe	7 (1.78)
	Villa de Garafía	2 (0.51)
**Educational level (n=379)**	No studies	10 (2.64)
	Elementary	88 (23.22)
	Secondary education	185 (48.81)
	University degree	96 (25.33)
**Employment status**	Active	238 (60.6)
	Inactive	155 (39.4)
**Occupation (n=388)**	Skilled agricultural. forestry and fishery workers	39 (10.05)
	Managers	10 (2.58)
	Elementary occupations	72 (18.56)
	Craft and related trades workers	22 (5.67)
	Plant and machine operators and assemblers	7 (1.80)
	Clerical support workers	36 (9.28)
	Professionals	44 (11.34)
	Technicians and associate professionals	38 (9.79)
	Service and sales workers	109 (28.09)
	Others out of classification	11 (2.84)
**Occupational exposure to toxic substances**	No	323 (82.19)
	Yes	70 (17.81)
**Type of toxic substances (n=63)**	Heavy metals	4 (6.35)
	Pesticides	14 (22.22)
	Radiation and radioactive materials	1 (1.59)

	Solvents and vapors	27 (42.86)
	Chemical mixture	17 (26.98)
**Years of occupational exposure to toxic substances; median (IQR) (n=58)**		15.5 (5-23)
**Years of occupational exposure to toxic substances (n=58)**	<15 años	25 (43.10)
	≥15 años	33 (56.90)
**Smoking exposure**	No	220 (55.98)
	Yes	173 (44.02)
**Years of exposure to smoking; median (IQR) (n=168)**		20 (10-30)
**Years of exposure to smoking (n=168)**	<20 años	65 (38.69)
	≥20 años	103 (61.31)
**Smoker status**	Current smoker	78 (19.85)
	Ex-smoker	95 (24.17)
	Never smoked	220 (55.98)
**Volcanic ash cleaning**	No	32 (8.14)
	Yes	361 (91.86)
**Cleaning location (n=361)**	Outdoor	59 (16.34)
	Indoor	11 (3.05)
	Both	291 (80.61)
Cleaning tools (n=361)	High (particle projection)	333 (92.24)
	Moderate (particle projection)	21 (5.82)
	Low (particle projection)	7 (1.94)
Cleaning frecuency (n=356)	≥1 once a day	186 (52.25)
	1-6 times per week	155 (43.54)
	Every 15 days/monthly	15 (4.21)
**Daily hours spent in outdoor environments**	No or <1 hour	41 (10.43)
	1-5 hours	150 (38.17)
	>5 hours	202 (51.4)
**Frequency of mask use outdoors (n=392)**	Always	330 (84.18)
	Mostly	43 (10.97)
	Rarely	14 (3.57)
	Never	5 (1.28)
**Frequency of protective eyeglasses use outdoors (n=392)**	Always	116 (29.59)
	Mostly	110 (28.06)
	Rarely	79 (20.15)
	Never	87 (22.19)

Abbreviations: ND: no data; NA no applied; IQR: Interquartile range. **Other specifications:** in cases where the number of observations does not correspond to the total sample size, the specific count used for the percentage calculations is explicitly indicated alongside the variable.

Table S2 Quantitative levels of inorganic elements in whole blood (ng/mL) among participants from the western region.

	Total sample (n=393)			
**Inorganic elements included in the ATSDR's priority pollutant list (2022)**	**Frequency of detection (%)**	**Mean (SD)**	**Median (IQR)**	**95%Confidence Interval (lower limit-upper limit)**
**Al (Aluminum)**	30.53	518.6 (949.59)	80.36 (39.42-180.76)	(346.95-690.24)
**As (arsenic)**	3.31	4.28 (3.86)	3.62 (0.63-6.87)	(1.94-6.61)
**Ba (Barium)**	21.88	4.76 (8.29)	2.28 (1.69-4.19)	(2.98-6.54)
**Be (Beryllium)**	14.76	1.43 (1.3)	1.29 (0.78-1.35)	(1.09-1.78)
**Cd (cadmium)**	45.80	0.46 (0.69)	0.23 (0.12-0.45)	(0.36-0.56)
**Co (Cobalt)**	44.02	0.26 (0.24)	0.21 (0.13-0.29)	(0.23-0.30)
**Cr (Chromium)**	4.07	10.95 (14.14)	5.89 (5.5-10.05)	(3.42-18.49)
**Cs (Cesium)**	95.42	1.77 (0.86)	1.66 (1.14-2.32)	(1.69-1.86)
**Cu (Copper)**	100.00	888.33 (189.32)	842.33 (777.62-954.77)	(869.56-907.11)
**Hg (mercury)**	98.73	3.87 (3.15)	3.13 (1.58-5.1)	(3.55-4.18)
**Mn (Manganese)**	96.44	7.87 (2.88)	7.48 (6.12-9.48)	(7.58-8.16)
**Ni (Nickel)**	40.46	10.96 (62.64)	3.86 (1.9-5.58)	(1.15-20.78)
**Pb (lead)**	92.11	7.73 (7.18)	6.04 (3.65-9.75)	(6.99-8.47)
**Sb (Antimony)**	8.65	11.39 (12.79)	4.55 (2.45-18.73)	(6.93-15.86)
**Se (Selenium)**	100.00	112.77 (30.04)	109.24 (94.06-126.07)	(109.79-115.75)
**Sr (Strontium)**	98.47	14.24 (6.08)	13.6 (10.48-17.05)	(13.63-14.85)
**Th (Thorium)**	14.76	0.59 (1.29)	0.12 (0.08-0.37)	(0.25-0.93)
**Tl (thallium)**	0.00	< LOQ	< LOQ	< LOQ
**U (Uranium)**	1.53	0.6 (0.55)	0.42 (0.15-1.2)	(0.03-1.18)
**V (Vanadium)**	2.80	1.27 (1.8)	0.58 (0.33-1.35)	(0.06-2.49)

**Other inorganic elements not included in the priority pollutant list**				
**Bi (bismuth)**	0.00	< LOQ	< LOQ	< LOQ
**Ce (cerium)**	15.01	0.15 (0.17)	0.1 (0.08-0.15)	(0.10-0.20)
**Dy (dysprosium)**	1.78	0.07 (0.02)	0.06 (0.06-0.09)	(0.06-0.09)
**Er (erbium)**	0.25	0.1 (-)	0.1 (0.1-0.1)	< LOQ
**Eu (europium)**	0.51	0.09 (0.04)	0.09 (0.06-0.12)	(-0.26-0.44)
**Gd (gadolinium)**	1.02	0.57 (0.59)	0.48 (0.07-1.06)	(-0.37-1.50)
**Ho (holmium)**	0.00	< LOQ	< LOQ	< LOQ
**In (indium)**	0.25	0.76 (-)	0.76 (0.76-0.76)	< LOQ
**La (lanthanum)**	7.38	0.14 (0.15)	0.09 (0.06-0.12)	(0.08-0.19)
**Lu (lutetium)**	0.25	0.05 (-)	0.05 (0.05-0.05)	< LOQ
**Nb (niobium)**	0.00	< LOQ	< LOQ	< LOQ
**Nd (neodimium)**	2.04	0.13 (0.09)	0.1 (0.06-0.17)	(0.05-0.20)
**Pr (praseodymium)**	0.25	0.05 (-)	0.05 (0.05-0.05)	< LOQ
**Pt (platinum)**	2.80	0.11 (0.06)	0.08 (0.07-0.11)	(0.06-0.15)
**Rb (Rubidium)**	100.00	1869.86 (381.97)	1848.83 (1621.31-2088.65)	(1831.98-1907.75)
**Ru (ruthenium)**	2.29	0.08 (0.04)	0.06 (0.05-0.08)	(0.05-0.11)
**Sm (samarium)**	3.56	0.08 (0.03)	0.07 (0.06-0.09)	(0.06-0.10)
**Sn (Tin)**	32.57	6.84 (5.25)	4.76 (2.87-10.28)	(5.92-7.76)
**Tb (terbium)**	0.00	< LOQ	< LOQ	< LOQ
**Ti (Titanium)**	69.72	8.26 (4.18)	7.22 (5.41-10.32)	(7.77-8.76)
**Tm (thulium)**	0.00	< LOQ	< LOQ	< LOQ
**Y (Ytrium)**	17.30	0.15 (0.12)	0.1 (0.07-0.19)	(0.12-0.18)
**Yb (ytterbium)**	1.27	0.08 (0.03)	0.07 (0.06-0.09)	(0.04-0.12)

8 **Abbreviations:** LOQ: limit of quantification; SD: standard deviation; IQR: Interquartile range.

Table S3 Quantitative levels of inorganic pollutants (ng/mL) in whole blood by sociodemographic characteristics among participants from the western region.

	**Inorganic elements**	**Cd**	**Hg**	**Pb**	**Mn**	**Co**	**Cu**	**Se**	**Al**	**Ti**	**Ni**	**Sr**	**Sn**	**Cs**	**Rb**
	**% of detection**	**46%**	**99%**	**92%**	**96%**	**44%**	**100%**	**100%**	**31%**	**70%**	**41%**	**99%**	**33%**	**95%**	**100%**
**Variables**		Median (IQR)	Median (IQR)	Median (IQR)	Median (IQR)	Median (IQR)	Median (IQR)	Median (IQR)	Median (IQR)	Median (IQR)	Median (IQR)	Median (IQR)	Median (IQR)	Median (IQR)	Median (IQR)
**Age category (years)**	≤50	0.18 (0.11-0.34)	2.52 (1.49-4.29) **	5.79 (3.21-9.43)	7.37 (5.82-9.65)	0.21 (0.13-0.31)	830.95 (769.41-936.97)	106.16 (92.64-125.51)	81.04 (37.42-189.18)	7.24 (5.41-10.07)	3.40 (1.84-5.10)	12.50 (9.51-15.26) ***	4.93 (2.23-11.85)	1.60 (1.08-2.24)	1827.54 (1612.93 - 2045.68)
	>50	0.25 (0.12-0.51)	3.60 (1.76-5.87)	6.42 (4.09-9.78)	7.53 (6.16-9.36)	0.22 (0.14-0.27)	855.81 (783.72-963.35)	113.75 (94.60-127.23)	79.67 (43.33-163.65)	7.19 (5.39-11.20)	4.34 (2.03-6.25)	14.89 (12.02 -18.80)	4.76 (3.25-7.14)	1.71 (1.22-2.35)	1867.65 (1621.31 - 2121.30)
**Sex**	Male	0.21 (0.12-0.47)	3.58 (1.59-5.86)	8.72 (5.09-13.11) ***	7.52 (6.19-9.27)	0.20 (0.13-0.27)	793.91 (740.04-852.86) ***	110.21 (95.35-127.45)	57.28 (32.81-169.82) *	7.49 (5.59-10.71)	2.68 (1.66-4.72) **	13.55 (10.53 -16.35)	4.27 (2.50-7.71)	1.65 (1.08-2.55)	1956.30 (1709.11 - 2198.33) ***
	Female	0.23 (0.12-0.44)	2.89 (1.57-4.75)	4.70 (3.01-7.23)	7.37 (5.83-10.10)	0.22 (0.13-0.31)	913.97 (820.57-1021.53)	108.31 (92.26-125.13)	100.07 (55.46-273.15)	7.05 (5.26-9.87)	4.40 (2.31-6.56)	13.84 (10.43 -17.66)	5.56 (4.01-10.94)	1.66 (1.16-2.22)	1767.39 (1559.30 - 2010.65)
**Educational level**	Elementary	0.34 (0.21-0.71) **	3.34 (1.53-5.60) **	6.56 (4.18-11.17)	7.94 (6.54-10.06)	0.23 (0.13-0.27)	845.35 (768.74-940.47)	108.16 (94.20-130.79)	96.65 (58.72-889.51)	7.40 (5.65-9.70)	3.84 (2.18-4.92)	13.55 (11.45 -17.71)	4.65 (2.93-6.25)	1.69 (1.23-2.21)	1888.20 (1655.75 - 2155.98)
	Secondary education	0.25 (0.12-0.49)	2.63 (1.52-4.30)	6.10 (3.78-9.75)	7.14 (5.72-9.30)	0.20 (0.13-0.28)	852.86 (783.02-969.66)	109.08 (93.08-124.21)	61.70 (36.52-372.85)	7.05 (5.04-10.13)	4.27 (1.90-6.42)	13.57 (10.25 -16.17)	6.12 (3.04-11.91)	1.61 (1.07-2.22)	1839.14 (1640.35 - 2045.68)
	University degree	0.17 (0.10-0.30)	3.98 (2.33-6.22)	5.79 (3.66-8.69)	7.51 (5.77-9.86)	0.22 (0.17-0.30)	840.03 (779.26-975.74)	113.29 (96.50-130.05)	100.33 (56.57-163.87)	6.88 (5.71-9.80)	3.01 (1.98-5.24)	13.94 (10.50 -17.00)	4.27 (2.37-10.82)	1.77 (1.22-2.58)	1810.02 (1566.42 - 2077.59)
	No studies	0.20 (0.15-0.23)	2.07 (1.71-5.63)	3.00 (2.25-7.71)	7.81 (6.84-10.17)	0.16 (0.09-0.21)	828.09 (797.35-874.24)	104.12 (85.72-118.23)	106.41 (61.36-4.146.92)	8.06 (3.97-15.14)	2.92 (1.74-5.32)	17.75 (12.85 - 28.25)	4.56 (3.97-5.18)	1.44 (1.28-1.52)	1779.21 (1609.13 - 2409.77)

**Employment status**	Active	0.19 (0.11-0.37) **	3.31 (1.71-5.41)	5.89 (3.68-9.41)	7.53 (6.09-9.72)	0.20 (0.13-0.29)	833.77 (778.40-941.32)	110.69 (94.51-126.94)	75.78 (37.42-189.18)	7.19 (5.41-10.29)	3.54 (1.84-5.24)	13.55 (10.43-16.37)	5.15 (2.56-10.93)	1.63 (1.09-2.24)	1838.37 (1622.34-2072.82)
	Inactive	0.25 (0.15-0.85)	2.89 (1.41-4.83)	6.31 (3.50-9.87)	7.38 (6.14-9.29)	0.23 (0.13-0.29)	863.51 (772.42-976.39)	107.44 (93.63-125.43)	92.90 (46.75-172.33)	7.31 (5.38-10.39)	3.93 (2.14-6.08)	13.82 (10.53-17.66)	4.60 (3.04-7.14)	1.68 (1.27-2.33)	1858.48 (1610.11-2121.30)
**Occupational exposure to toxic substances**	No	0.22 (0.12-0.45)	3.24 (1.65-5.14)	5.84 (3.54-9.07)	7.48 (6.08-9.46)	0.21 (0.14-0.29)	852.86 (780.51-963.35) *	110.29 (94.40-126.63)	81.04 (40.08-169.82)	7.18 (5.41-10.13)	3.93 (1.90-5.91)	13.78 (10.50-17.10)	4.65 (2.97-7.86)	1.73 (1.22-2.35) *	1837.90 (1609.13-2065.42)
	Yes	0.25 (0.12-0.45)	2.50 (1.41-5.01)	6.93 (3.99-10.84)	7.70 (6.14-9.74)	0.24 (0.12-0.35)	824.69 (762.03-915.03)	106.61 (92.03-125.06)	75.78 (36.26-387.60)	7.55 (5.36-10.96)	2.68 (1.96-4.27)	13.30 (10.22-16.12)	6.22 (2.65-14.11)	1.51 (1.04-1.89)	1909.06 (1656.38-2144.30)
**Type of toxic substances**	Chemical mixture	0.16 (0.12-0.39)	2.56 (1.72-4.56)	7.46 (3.50-9.39)	8.03 (6.19-9.27)	0.12 (0.08-0.28)	827.10 (762.29-900.88)	98.97 (91.76-117.77)	43.92 (38.71-372.85)	6.06 (4.12-13.08)	4.10 (3.54-4.72)	12.28 (10.02-15.95)	15.82 (9.56-16.99)	0.83 (0.43-1.61)	1892.25 (1709.23-2151.49)
	Heavy metals	0.28 (0.10-0.45)	1.92 (1.68-2.80)	10.00 (5.32-14.09)	7.34 (5.35-8.64)	0.23 (0.19-0.27)	755.30 (683.08-1.157.31)	103.65 (96.89-122.58)	28.06 (28.06-28.06)	8.94 (6.53-14.81)	1.15 (0.94-1.35)	11.90 (8.60-13.51)	NA	1.34 (1.20-1.94)	1768.61 (1641.61-1972.75)
	Pesticides	0.23 (0.12-0.52)	4.63 (1.56-7.72)	9.83 (6.44-10.84)	7.94 (6.87-9.28)	0.24 (0.11-0.25)	802.87 (685.74-931.72)	121.33 (99.25-130.77)	2.029.70 (114.46-2.046.19)	9.06 (6.50-13.66)	4.02 (2.34-5.58)	18.19 (10.09-26.05)	4.28 (2.81-10.24)	1.55 (1.05-1.89)	2074.05 (1863.62-2574.20)
	Radiation and radioactive materials	NA	2.24 (2.24-2.24)	NA	4.98 (4.98-4.98)	NA	1.639.57 (1.639.57-1.639.57)	112.06 (112.06-112.06)	93.81 (93.81-93.81)	NA	NA	5.72 (5.72-5.72)	NA	NA	1551.51 (1551.51-1551.51)
	Solvents and vapours	0.33 (0.19-0.50)	2.47 (1.21-5.28)	6.73 (3.95-14.70)	7.18 (5.95-10.34)	0.31 (0.20-0.47)	794.79 (746.82-893.39)	100.17 (88.23-120.07)	59.30 (32.81-1885.53)	7.10 (5.88-9.64)	2.18 (1.84-4.18)	13.88 (13.04-17.37)	3.22 (1.95-6.24)	1.51 (1.07-1.97)	1925.87 (1652.78-2198.33)
**Years of occupational exposure to toxic substances**	<15 years	0.33 (0.12-0.44)	1.88 (1.21-2.69) *	6.60 (3.39-9.39)	6.85 (5.95-9.27) *	0.21 (0.12-0.27)	849.24 (794.79-957.80) *	99.25 (89.88-117.77)	84.80 (52.92-243.65)	7.62 (5.92-9.23)	4.02 (2.18-4.27)	13.76 (11.09-17.91)	7.10 (2.09-16.59)	1.54 (1.17-1.82)	1881.25 (1709.23-2141.86)
	≥15 years	0.21 (0.13-0.50)	3.98 (2.00-6.32)	8.79 (5.38-14.13)	8.55 (6.82-10.48)	0.27 (0.11-0.48)	790.40 (711.20-838.32)	108.85 (97.90-126.07)	100.49 (28.06-2.046.19)	8.65 (5.08-12.69)	2.16 (1.56-4.72)	13.88 (10.32-18.23)	6.24 (3.68-11.32)	1.48 (1.05-1.83)	1991.10 (1760.41-2222.62)

**Smoking exposure**	No	0.14 (0.10-0.25) ***	3.12 (1.54-5.21)	4.99 (3.16-8.17) ***	7.65 (6.28-9.93) *	0.22 (0.15-0.30)	854.02 (778.01-972.25)	110.59 (94.18-127.34)	79.67 (40.08-123.26)	7.19 (5.39-10.13)	3.89 (1.93-5.85)	13.22 (10.37-16.28)	5.24 (3.41-11.39)	1.65 (1.09-2.33)	1830.64 (1587.63-2064.24)
	Yes	0.34 (0.19-0.78)	3.13 (1.60-4.94)	7.57 (4.70-12.32)	7.34 (5.81-9.06)	0.19 (0.11-0.29)	831.25 (774.12-940.72)	108.39 (93.90-125.50)	92.97 (37.10-1.329.38)	7.30 (5.44-10.89)	3.83 (1.84-5.39)	13.98 (10.82-17.72)	4.31 (2.55-7.42)	1.67 (1.22-2.24)	1893.61 (1645.94-2128.14)
**Smoker status**	Current smoker	0.55 (0.29-1.04) ***	2.51 (1.46-4.33)	7.54 (4.72-13.25) ***	6.77 (5.81-8.77)	0.18 (0.11-0.29)	818.37 (764.52-929.48)	101.15 (91.42-119.45)	96.52 (37.10-1.885.53)	7.53 (5.51-9.48)	3.25 (1.84-6.08)	13.86 (10.53-17.37)	3.93 (2.10-6.31)	1.60 (1.19-2.22)	1958.75 (1673.77-2101.57)
	Ex-smoker	0.22 (0.11-0.34)	3.69 (1.96-5.63)	7.62 (4.70-11.32)	7.44 (5.85-9.31)	0.21 (0.12-0.28)	842.33 (779.72-960.00)	111.58 (98.66-127.07)	65.13 (38.71-732.49)	6.83 (5.40-12.67)	3.91 (2.21-5.08)	14.12 (11.34-18.45)	4.37 (3.29-8.63)	1.70 (1.24-2.40)	1813.83 (1622.42-2155.41)
	Never smoked	0.14 (0.10-0.25)	3.12 (1.54-5.21)	4.99 (3.16-8.17)	7.65 (6.28-9.93)	0.22 (0.15-0.30)	854.02 (778.01-972.25)	110.59 (94.18-127.34)	79.67 (40.08-123.26)	7.19 (5.39-10.13)	3.89 (1.93-5.85)	13.22 (10.37-16.28)	5.24 (3.41-11.39)	1.65 (1.09-2.33)	1830.64 (1587.63-2064.24)
**Years of exposure to smoking**	<20 years	0.29 (0.10-0.65)	3.13 (1.93-3.98)	6.60 (4.03-9.58) *	7.38 (5.78-9.37)	0.17 (0.09-0.26)	827.10 (765.44-905.82)	113.09 (92.65-125.50)	84.37 (34.95-156.72)	7.56 (5.32-11.17)	4.18 (2.31-5.09)	13.56 (10.25-15.95) *	4.40 (2.08-9.56)	1.49 (1.02-2.15)	1744.94 (1622.34-2155.41)
	≥20 years	0.44 (0.22-0.91)	3.12 (1.31-5.56)	8.41 (5.84-14.34)	7.26 (6.08-8.64)	0.22 (0.16-0.33)	838.32 (779.72-957.80)	106.92 (94.26-126.07)	93.48 (40.99-1885.53)	7.15 (5.51-10.89)	3.14 (1.81-5.63)	14.42 (11.62-19.39)	4.27 (2.50-6.24)	1.70 (1.27-2.39)	1965.16 (1677.86-2128.14)

Abbreviations: NA: not applicable. **p value meaning:** *: <0.05; **:<0.01; ***:<0.001. **Other specifications:** Dichotomous variables were analysed by Mann-Whitney U test; Polytomous variables were analysed by Kruskal-Wallis test.

Table S4 Quantitative levels of Trace elements (ng/mL) in whole blood of the participants from the western region in relation to the level of exposure to Tajogaite volcano during eruption.

	**Inorganic elements**	**Cd**	**Hg**	**Pb**	**Mn**	**Co**	**Cu**	**Se**	**Al**	**Ti**	**Ni**	**Sr**	**Sn**	**Cs**	**Rb**
	**% of detection**	**46%**	**99%**	**92%**	**96%**	**44%**	**100%**	**100%**	**31%**	**70%**	**41%**	**99%**	**33%**	**95%**	**100%**
**Variables**		Median (IQR)	Median (IQR)	Median (IQR)	Median (IQR)	Median (IQR)	Median (IQR)	Median (IQR)	Median (IQR)	Median (IQR)	Median (IQR)	Median (IQR)	Median (IQR)	Median (IQR)	Median (IQR)
**Distance (usual residence) to volcano (km)**	<6.5	0.23 (0.12-0.48)	3.24 (1.75-4.84)	6.38 (3.67-10.20)	7.61 (6.21-9.76)	0.23 (0.16-0.30)	845.46 (781.52-946.44)	107.93 (90.47-125.51) *	107.82 (49.45-323.00) *	7.60 (5.70-11.40) *	3.68 (1.82-5.24)	13.76 (9.98-16.96)	4.37 (2.39-7.00) **	1.54 (1.06-2.24)	1849.40 (1637.95-2121.30)
	≥6.5	0.23 (0.12-0.44)	3.07 (1.50-5.28)	5.85 (3.52-8.94)	7.29 (5.83-9.27)	0.20 (0.12-0.27)	839.18 (771.31-966.81)	111.51 (96.57-129.42)	55.33 (36.81-100.41)	6.88 (5.04-9.36)	4.02 (2.38-6.56)	13.47 (10.69-17.21)	5.85 (3.63-12.22)	1.73 (1.23-2.33)	1844.72 (1593.16-2054.37)
**Volcanic ash cleaning**	No	0.23 (0.17-0.43)	3.85 (1.57-6.43)	6.75 (3.00-11.83)	6.98 (5.95-10.19)	0.20 (0.13-0.23)	809.56 (777.16-896.52)	107.38 (92.52-122.81)	103.61 (30.62-2295.76)	7.05 (5.80-11.06)	2.09 (1.34-2.77) *	15.86 (12.53-18.90) *	3.58 (1.93-4.60)	1.54 (0.97-2.64)	1783.51 (1558.16-2011.63)
	Yes	0.22 (0.11-0.45)	3.12 (1.61-4.92)	5.92 (3.66-9.59)	7.49 (6.12-9.48)	0.22 (0.13-0.29)	845.23 (777.62-960.00)	109.36 (94.26-126.20)	79.67 (40.08-169.82)	7.24 (5.39-10.32)	3.93 (1.98-5.70)	13.55 (10.32-16.64)	4.93 (2.89-10.82)	1.67 (1.16-2.24)	1852.29 (1622.34-2089.01)
**Cleaning location**	Both	0.22 (0.12-0.45)	3.23 (1.68-4.85)	5.89 (3.69-9.60)	7.53 (6.13-9.48)	0.21 (0.13-0.29)	849.24 (778.40-970.87)	109.69 (94.15-127.07)	90.84 (41.29-180.76)	7.18 (5.39-10.71)	3.91 (1.98-5.70)	13.62 (10.40-16.63)	4.76 (2.89-9.56)	1.66 (1.16-2.22)	1849.40 (1637.95-2090.92)
	Outdoor	0.19 (0.09-0.34)	2.72 (1.46-5.28)	6.21 (3.47-9.78)	7.32 (6.05-9.76)	0.25 (0.15-0.30)	834.25 (764.50-917.75)	107.50 (94.26-120.38)	68.85 (37.42-163.87)	7.90 (5.92-10.29)	4.19 (1.84-5.58)	13.09 (10.26-16.63)	6.10 (2.56-11.32)	1.65 (1.08-2.32)	1867.94 (1612.60-

															2088.65)
	Indoor	1.89 (0.19-3.51)	2.12 (1.10-4.03)	5.14 (3.75-8.51)	6.84 (5.58-8.82)	0.21 (0.13-0.29)	973.63 (816.15-1103.68)	112.06 (80.12-145.78)	51.10 (29.47-93.81)	5.81 (5.01-8.60)	5.10 (4.83-5.94)	10.67 (9.16-16.64)	13.12 (6.84-19.40)	2.02 (1.64-2.58)	1906.68 (1547.74-2225.05)
Cleaning tools	High (particle projection)	0.23 (0.12-0.47)	3.13 (1.54-5.14)	6.04 (3.72-9.76)	7.51 (6.08-9.43)	0.22 (0.13-0.29)	845.46 (776.81-963.97)	110.16 (94.30-126.20)	75.78 (38.71-169.82)	7.22 (5.30-10.47)	4.18 (2.14-5.80)	13.56 (10.25-17.03)	5.11 (2.85-10.93)	1.68 (1.16-2.29)	1852.29 (1622.42-2088.65)
	Moderate (particle projection)	0.23 (0.13-0.81)	3.57 (2.07-3.84)	4.05 (1.85-6.28)	6.76 (3.65-7.27)	0.20 (0.14-0.27)	882.87 (820.59-1270.37)	105.09 (103.78-107.30)	116.39 (85.57-2417.46)	6.55 (5.59-7.86)	1.92 (1.40-3.32)	13.84 (9.51-16.67)	3.04 (3.04-3.04)	1.50 (0.81-2.09)	1559.70 (1256.44-1979.73)
	Low (particle projection)	0.15 (0.11-0.25)	2.33 (1.68-4.77)	5.21 (3.43-7.57)	7.53 (6.54-9.89)	0.25 (0.18-0.29)	833.30 (785.70-899.40)	102.52 (91.72-129.51)	112.17 (75.72-538.38)	7.94 (6.48-9.47)	3.69 (1.88-7.42)	13.04 (11.57-15.15)	4.82 (4.76-5.01)	1.54 (1.22-1.96)	1902.42 (1682.80-2142.61)
Cleaning frequency	>=1 once a day	0.25 (0.12-0.50)	3.16 (1.64-5.17)	5.79 (3.50-9.75)	7.38 (6.01-9.34)	0.20 (0.13-0.28)	874.27 (788.01-1006.37) **	111.19 (92.64-132.13)	83.49 (41.60-189.18)	6.98 (5.28-9.75)	4.18 (2.14-6.68)	13.57 (10.27-16.28)	5.13 (3.04-11.71)	1.77 (1.32-2.37) *	1840.03 (1640.35-2067.41)
	1-6 times per week	0.19 (0.11-0.43)	3.12 (1.70-4.83)	6.04 (3.66-9.04)	7.73 (6.21-9.81)	0.23 (0.15-0.30)	834.38 (769.41-906.15)	109.58 (96.64-123.37)	79.70 (38.71-163.87)	7.28 (5.36-10.29)	3.93 (1.98-5.14)	13.56 (10.49-16.67)	4.55 (2.71-7.52)	1.55 (1.05-2.15)	1867.94 (1574.16-2096.54)
	Every 15 days/monthly	0.18 (0.08-0.34)	3.07 (1.29-4.56)	6.91 (4.64-11.03)	6.49 (5.07-9.66)	0.09 (0.08-0.26)	769.25 (740.04-932.50)	103.78 (95.33-127.23)	62.49 (52.68-116.39)	11.66 (8.01-12.95)	2.83 (1.47-4.19)	11.11 (7.13-14.45)	3.98 (2.86-11.18)	1.52 (0.82-2.32)	1853.22 (1652.78-2255.40)
**Daily hours spent in outdoor**	No or <1 hour	0.35 (0.19-1.16)	2.86 (1.26-5.63)	5.03 (2.57-	6.91 (6.12-9.48)	0.20 (0.10-0.24)	883.61 (820.59 -	99.28 (80.12-	93.48 (46.54-172.33)	6.48 (4.62-9.47)	3.91 (1.98-6.07)	13.24 (10.25	5.10 (2.60-10.24)	1.50 (1.21-2.29)	1775.27 (1679.86-

**environments**				8.00) **			987.01) **	115.56) **				- 16.96)			1927.04)
	1-5 hours	0.23 (0.10-0.34)	3.27 (1.56-4.97)	5.52 (3.39-8.56)	7.45 (5.82-9.92)	0.21 (0.13-0.30)	866.66 (783.96-973.63)	108.92 (91.50-126.94)	88.78 (39.05-169.82)	8.23 (5.85-11.77)	4.29 (1.90-6.08)	14.10 (10.50-18.23)	4.57 (2.41-7.33)	1.74 (1.15-2.33)	1838.37 (1603.81-2089.01)
	>5 hours	0.21 (0.12-0.47)	3.07 (1.71-4.98)	6.56 (4.14-10.71)	7.50 (6.16-9.32)	0.22 (0.14-0.29)	826.93 (770.53-916.70)	112.69 (97.52-127.07)	73.04 (39.25-281.02)	7.05 (5.17-9.48)	3.46 (1.90-5.09)	13.47 (10.49-16.17)	4.97 (3.15-11.07)	1.61 (1.09-2.37)	1879.15 (1645.94-2096.54)
**Frequency of mask use outdoors**	Always	0.22 (0.12-0.42)	3.09 (1.68-4.86)	5.89 (3.53-9.04)	7.51 (6.16-9.75)	0.22 (0.13-0.28)	845.39 (773.55-963.30)	109.07 (94.15-125.13)	85.57 (40.99-163.87)	7.46 (5.42-10.85)	3.91 (1.98-5.54)	13.57 (10.50-16.67)	4.75 (2.90-9.90)	1.62 (1.13-2.33)	1851.51 (1634.20-2090.92)
	Mostly	0.44 (0.12-0.71)	4.10 (1.46-6.22)	6.55 (3.75-12.85)	7.18 (5.78-8.61)	0.24 (0.15-0.35)	845.23 (780.51-906.15)	110.50 (95.19-133.87)	49.85 (31.85-1.934.01)	6.87 (5.51-9.48)	3.93 (2.34-5.60)	13.76 (10.45-17.10)	6.87 (2.07-9.56)	1.73 (1.35-2.15)	1837.90 (1621.31-2046.14)
	Rarely	0.14 (0.10-0.45)	3.53 (1.31-5.44)	7.50 (4.16-9.34)	7.68 (5.83-9.40)	0.19 (0.16-0.20)	822.33 (769.41-871.69)	121.98 (92.65-144.26)	384.38 (32.16-1030.94)	5.97 (4.62-9.23)	1.53 (1.35-6.08)	13.70 (9.89-19.47)	8.25 (2.54-12.47)	1.39 (1.25-2.34)	1768.61 (1486.27-2175.93)
	Never	0.28 (0.09-0.47)	1.98 (1.53-5.98)	5.68 (3.94-13.50)	5.76 (4.78-6.09)	0.19 (0.06-0.54)	795.15 (790.68-874.32)	117.81 (111.43-138.64)	27.03 (27.03-27.03)	6.92 (6.63-7.24)	1.96 (1.96-1.96)	13.19 (10.16-17.21)	NA	2.00 (1.67-2.15)	1937.81 (1700.56-2198.33)
**Frequency of protective eyeglasses use outdoors**	Always	0.25 (0.12-0.50)	3.37 (1.72-4.92)	5.92 (3.81-9.25)	7.57 (6.18-9.53)	0.20 (0.13-0.27)	860.54 (787.82-942.58)	109.17 (91.05-125.60)	99.81 (39.78-169.82)	7.00 (5.08-9.70)	3.26 (1.76-4.97)	13.66 (10.85-16.65)	4.82 (2.41-9.56)	1.61 (1.30-2.15)	1811.39 (1642.43-1949.91)

	Mostly	0.18 (0.12-0.31)	3.12 (1.65-5.26)	6.00 (3.52-10.07)	7.65 (6.28-9.76)	0.24 (0.17-0.30)	834.31 (769.25-942.05)	110.23 (97.96-125.43)	68.85 (35.04-538.38)	7.38 (5.51-11.61)	4.18 (1.90-6.30)	14.01 (11.34-17.35)	5.12 (3.79-13.75)	1.73 (1.16-2.40)	1900.37 (1621.31-2096.54)
	Rarely	0.25 (0.12-0.45)	3.12 (1.50-5.88)	6.49 (3.87-9.83)	7.36 (5.99-9.82)	0.18 (0.12-0.33)	831.49 (773.10-994.43)	114.99 (92.65-132.13)	103.61 (46.54-658.03)	7.45 (5.84-9.95)	4.31 (2.34-6.42)	13.62 (10.25-17.03)	4.75 (2.50-11.61)	1.62 (1.16-2.36)	1947.44 (1617.83-2168.94)
	Never	0.26 (0.12-0.52)	2.77 (1.53-4.86)	5.56 (3.19-9.34)	7.11 (5.82-9.06)	0.23 (0.13-0.31)	845.46 (777.37-957.80)	106.92 (94.30-121.06)	61.36 (28.50-99.56)	7.32 (5.41-10.50)	3.20 (1.84-5.09)	13.19 (9.78-15.71)	4.33 (2.54-6.87)	1.59 (1.02-2.21)	1862.67 (1559.30-2128.14)

Abbreviations: NA: not applicable. **p value meaning:** *: <0.05; **:<0.01; ***:<0.001. **Other specifications:** Dichotomous variables were analysed by Mann-Whitney U test; Polytomous variables were analysed by Kruskal-Wallis test.
